# Stable Gastric Pentadecapeptide BPC 157 Therapy: Effect on Reperfusion Following Maintained Intra-Abdominal Hypertension (Grade III and IV) in Rats

**DOI:** 10.3390/ph16111554

**Published:** 2023-11-02

**Authors:** Marijan Tepes, Ivan Krezic, Hrvoje Vranes, Ivan Maria Smoday, Luka Kalogjera, Helena Zizek, Vlasta Vukovic, Katarina Oroz, Katarina Kasnik Kovac, Zrinko Madzar, Mislav Rakic, Blazenka Miskic, Suncana Sikiric, Ivan Barisic, Sanja Strbe, Marko Antunovic, Luka Novosel, Ivana Kavelj, Josipa Vlainic, Ivan Dobric, Mario Staresinic, Anita Skrtic, Sven Seiwerth, Alenka Boban Blagaic, Predrag Sikiric

**Affiliations:** 1 Department of Pharmacology, School of Medicine, University of Zagreb, 10000 Zagreb, Croatia; mtepes@gmail.com (M.T.); ivankrezic94@gmail.com (I.K.); hrvoje.vranes@gmail.com (H.V.); ivansmoday1@gmail.com (I.M.S.); lkalogjera9@gmail.com (L.K.); zizekhelena@gmail.com (H.Z.); vukovic.vlasta1@gmail.com (V.V.); oroz.kat@hotmail.com (K.O.); kkasnik@gmail.com (K.K.K.); zrinko.madzar@gmail.com (Z.M.); inbarisic@gmail.com (I.B.); strbes@gmail.com (S.S.); novosel0701@gmail.com (L.N.); ivana.kavelj@gmail.com (I.K.); abblagaic@mef.hr (A.B.B.); 2 Department of Clinical Medicine, Faculty of Dental Medicine and Health Osijek, 31000 Osijek, Croatia; miskicblazenka@gmail.com; 3 PhD Program Translational Research in Biomedicine-TRIBE, School of Medicine, University of Split, 21000 Split, Croatia; 4 Department of Abdominal Surgery, Clinical Hospital Dubrava, 10040 Zagreb, Croatia; mislav78@gmail.com; 5 Department of Pathology, School of Medicine, University of Zagreb, 10000 Zagreb, Croatia; suncanasikiric@gmail.com (S.S.); sven.seiwerth@mef.hr (S.S.); 6 Laboratory for Advanced Genomics, Division of Molecular Medicine, Institute Ruder Boskovic, 10000 Zagreb, Croatia; josipa.vlainic@irb.hr; 7 Department of Surgery, School of Medicine, University of Zagreb, 10000 Zagreb, Croatia; ivandobricmd@gmail.com

**Keywords:** prime acute abdominal compartment, occlusion/occlusion-like syndrome, reperfusion, stable gastric pentadecapeptide BPC 157 therapy

## Abstract

Given in reperfusion, the use of stable gastric pentadecapeptide BPC 157 is an effective therapy in rats. It strongly counteracted, as a whole, decompression/reperfusion-induced occlusion/occlusion-like syndrome following the worst circumstances of acute abdominal compartment and intra-abdominal hypertension, grade III and grade IV, as well as compression/ischemia-occlusion/occlusion-like syndrome. Before decompression (calvariectomy, laparotomy), rats had long-lasting severe intra-abdominal hypertension, grade III (25 mmHg/60 min) (i) and grade IV (30 mmHg/30 min; 40 mmHg/30 min) (ii/iii), and severe occlusion/occlusion-like syndrome. Further worsening was caused by reperfusion for 60 min (i) or 30 min (ii/iii). Severe vascular and multiorgan failure (brain, heart, liver, kidney, and gastrointestinal lesions), widespread thrombosis (peripherally and centrally) severe arrhythmias, intracranial (superior sagittal sinus) hypertension, portal and caval hypertension, and aortal hypotension were aggravated. Contrarily, BPC 157 therapy (10 µg/kg, 10 ng/kg sc) given at 3 min reperfusion times eliminated/attenuated venous hypertension (intracranial (superior sagittal sinus), portal, and caval) and aortal hypotension and counteracted the increases in organ lesions and malondialdehyde values (blood ˃ heart, lungs, liver, kidney ˃ brain, gastrointestinal tract). Vascular recovery promptly occurred (i.e., congested inferior caval and superior mesenteric veins reversed to the normal vessel presentation, the collapsed azygos vein reversed to a fully functioning state, the inferior caval vein–superior caval vein shunt was recovered, and direct blood delivery returned). BPC 157 therapy almost annihilated thrombosis and hemorrhage (i.e., intracerebral hemorrhage) as proof of the counteracted general stasis and Virchow triad circumstances and reorganized blood flow. In conclusion, decompression/reperfusion-induced occlusion/occlusion-like syndrome counteracted by BPC 157 therapy in rats is likely for translation in patients. It is noteworthy that by rapidly counteracting the reperfusion course, it also reverses previous ischemia-course lesions, thus inducing complete recovery.

## 1. Introduction

We attempt to resolve in a particular cytoprotection way (i.e., endothelium functioning or malfunctioning) [1,2,3], the threat of reperfusion after the prime acute abdominal compartment and intra-abdominal hypertension grade III and grade IV. Likewise, we would emphasize the dangerous course of huge vascular and multiorgan failure in severe decompression/reperfusion-occlusion/occlusion-like syndrome and its resolution in rats by introducing the stable gastric pentadecapeptide BPC 157 therapy as a cytoprotective agent [1,2,3,4,5,6,7] (i.e., activation of azygos vein direct blood flow delivery) [8,9,10,11,12,13,14,15,16,17,18,19,20,21,22,23,24,25], which is given during reperfusion.

This novel resolution of the reperfusion threat could provide a particular new therapy point, in particular, given the recent findings on mechanical compression–ischemia showing that rats could sustain severe intra-abdominal hypertension grade III and grade IV and resolve severe vascular and multiorgan failure and major occlusion/occlusion-like syndrome without major harm after receiving BPC 157 therapy [8]. That, among others, included “bypassing key”, as a particular cytoprotection endothelium recovery, activation of the azygos vein pathway, and direct blood flow delivery as a rapid rescue [8] (as in other occlusion/occlusion-like syndromes [9,10,11,12,13,14,15,16,17,18,19,20,21,22,23,24,25]), and these beneficial findings together could indicate a particular way to approach the issue of intra-abdominal hypertension in the resolving therapy. As a possible translation for critically ill patients, note that when not promptly recognized and treated, intra-abdominal hypertension leads to abdominal compartment syndrome, multiorgan dysfunction syndrome, and death [26,27,28,29]. In addition, as stated, abdominal compartment syndrome is not a disease and, as such, it can have many causes and can develop within many disease processes [29], and thereby, as such, it may be at best approached using some of the general concepts, such as cytoprotection [30,31,32,33,34], which would offer particular solutions via cytoprotection agents’ application.

From a general conceptual point of the cytoprotection view recently reviewed [30,31,32,33,34], and in a recent series of reports [8,9,10,11,12,13,14,15,16,17,18,19,20,21,22,23,24,25], we reported occlusion/occlusion-like syndromes in rats [8,9,10,11,12,13,14,15,16,17,18,19,20,21,22,23,24,25]. This was done in an attempt to extend the original background of the stomach cytoprotection concept (based on the meaningful significance of alcohol stomach lesions, induction, and prevention) [35,36], Robert’s (epithelium protection) [35] and Szabo’s (endothelium protection) [36] intended for a large generalization. These occlusion/occlusion-like syndromes [8,9,10,11,12,13,14,15,16,17,18,19,20,21,22,23,24,25] highlight the methodology advantage of the demonstration of cytoprotection and endothelium malfunctioning (cause–consequence injurious course, occurring peripherally and centrally upon the initiation of noxious procedures or agents’ administration). Vice versa, it highlights therapy proof (endothelium maintenance/recovery to the activation of collateral pathways as key for a pleiotropic beneficial effect of cytoprotective agents that BPC 157 therapy might rapidly induce) [8,9,10,11,12,13,14,15,16,17,18,19,20,21,22,23,24,25]. These were rats with permanent major vessel occlusions, arteries, and veins, peripherally and centrally [9,10,11,12,13,14,15,16,17], who underwent similar noxious procedures or the application of agents [8,18,19,20,21,22,23,24,25] that all severely impacted endothelium function. In particular, this was the intra-abdominal hypertension occlusion/occlusion-like syndrome, grade III and grade IV, and nearly fatal ischemia (note: the prime abdominal compartment compression has multiple occlusion syndrome [8]). These were severe vascular and multiorgan failures (i.e., severe lesions in the brain (intracerebral, intraventricular hemorrhage) and heart (congestion, myocardial infarction, severe arrhythmias) and congestion/hemorrhage in the lungs, liver, kidney, and gastrointestinal tract). Major vessels had failed; there was widespread thrombosis in the veins and arteries, and stasis; there were advanced Virchow triad circumstances, peripherally and centrally; and there were intracranial (superior sagittal sinus), portal, and caval hypertension, and aortal hypotension [8,9,10,11,12,13,14,15,16,17,18,19,20,21,22,23,24,25], all as occlusion/occlusion-like syndromes attenuated/eliminated by BPC 157 therapy [8,9,10,11,12,13,14,15,16,17,18,19,20,21,22,23,24,25]. As emphasized, with such intra-abdominal hypertension, grade III- and grade IV-induced occlusion/occlusion-like syndrome [8] was worse and more severe than other occlusion/occlusion-like syndromes [9,10,11,12,13,14,15,16,17,18,19,20,21,22,23,24,25], and, thereby, almost-complete annihilation/elimination by BPC 157 therapy (i.e., activation of collateral pathways, or azygos vein direct blood flow delivery) may be quite indicative in these particular conditions [8]. Note: given the overall compression, the resolving of the ischemia of the prime abdominal compartment compression requires the multiple occlusion syndromes [8] to be simultaneously resolved within the therapy effect. Conceptually, this means the realization of the therapy effect at the multiple locations affected by the severe compression (intra-abdominal hypertension occlusion/occlusion-like syndrome grade III and grade IV), given that the whole syndrome was counteracted as a whole [8]. Thereby, via BPC 157 therapy, an even more demanding therapy effect than that in other occlusion/occlusion-like syndromes [9,10,11,12,13,14,15,16,17,18,19,20,21,22,23,24,25]—that BPC 157 therapy has consistently counteracted—can be promptly achieved [8]. On the other hand, this may show additional therapy potential for the activation of collateral pathways, i.e., azygos vein direct blood flow delivery, “bypassing key” that can be equally resolved after severe ischemic conditions (compression) [9,10,11,12,13,14,15,16,17,18,19,20,21,22,23,24,25] are instantly replaced by even more harmful reperfusion conditions (decompression) and post-decompression time.

Therefore, such therapy in the post-decompression time should resolve the worsening complexity of the advanced occlusion/occlusion-like syndrome. Antecedent to therapy, there is a syndrome that barely survives by itself (i.e., severe bradycardia and ST elevation until asystole) during ischemia and compression and the consequent disturbances, thrombosis, stasis, vascular and multiorgan failure, and lesions, as mentioned before [8]. As such [8], it has to be cured in reperfusion using the therapy even in the worst condition that would occur in reperfusion, arising simultaneously from many sides after the end of compression, anticipating the imminent aggravation.

Thus, a strong curative potential option in advanced injury courses should be to re-establish the significance of the therapy using cytoprotection agents that they have to have, as the concept has claimed [35] (while standard agents’ effectiveness only before injury is limited in application [35]). Conceptually, this may be stable gastric pentadecapeptide BPC 157 (note: BPC 157 was used in an ulcerative colitis clinical trial, phase II, and in toxicology studies, a lethal dose (LD1) could be not achieved) [1,2,3,30,31,32,33,34]. Conceptually for the stomach cytoprotection concept, native and stable in human gastric juice (i.e., more than 24 h), as a native cytoprotection mediator maintaining gastrointestinal tract integrity may translate into the particular therapy of other tissues and may be easily applied. In addition, in the cytoprotection concept [35], the cells afforded by the cytoprotection agent should equally resist noxious events, whether ischemia or reperfusion. Note that BPC 157, given in the reperfusion, showed a strong therapy effect in rats with the Pringle maneuver [10], stroke [17], spinal cord compression [37,38], and ischemic/reperfusion ulcerative colitis [39]. There, in studies with one prime target [10,17,37,38,39], there is the removal of the particular clamping (spinal cord) [37,38] and the reopening of the particular vessel(s) (i.e., Pringle maneuver, stroke, and ischemic/reperfusion ulcerative colitis) [10,17,39]. Note that such therapy, when given in these circumstances, thereafter means resolved reperfusion consequences occurring from one major prime target while leaving the therapy effect to be demonstrated on reperfusion that is going on from a large number of sites that would generate reperfusion in post-decompression time in rats that have intra-abdominal hypertension. On the other hand, these procedures, taken together [10,17,37,38,39], can indicate the functioning against reperfusion injuries from many sites as can be expected in post-decompression time in rats with previous severe intra-abdominal hypertension and compression.

It is likely that such commonality in the responses, with diverse noxious events [8,9,10,11,12,13,14,15,16,17,18,19,20,21,22,23,24,25] (as the cytoprotection concept posits for cytoprotective agents [35,36]), can suggest the equal efficacy of this therapy that should be demonstrated in the later course as well [6,30,31,32,33,34]. This vascular recovery potential [6,30,31,32,33,34] “bypassing key”, the activation of the collaterals depending on the given injury [6,30,31,32,33,34], has been supported by its special interaction with essential systems in cytoprotection, the prostaglandin system (counteraction of all adverse effects of non-steroidal anti-inflammatory drugs (NSAIDs)) [40], and then a nitric oxide (NO) system [41,42,43,44] as a whole. In support, BPC 157 therapy has a close interaction with NO synthases. As recently reviewed [30,31] in a series of very distinctive disturbances models, BPC 157 induced release of the NO by its own [43,44], counteraction of the adverse effect of NOS-blockade (i.e., hypertension, pro-thrombotic effect) or NOS-over-stimulation (i.e., hypotension, anti-thrombotic effect) [43,45], maintained thrombocytes function [45,46,47] and many molecular pathways [6,48,49,50,51,52,53,54,55,56,57,58], controlling vasomotor tone and the activation of Src-Caveolin-1-eNOS pathway [48,49]. There, in the vessel wall, the rapid change in the lipid contents and protein secondary structure conformation, produced instantly via BPC 157 therapy [59] (Fourier transform infrared spectroscopy), supported the vessel function even in the worst circumstances. All these might be its modulatory effects. Namely, acting also as a free radical scavenger [6,56,60,61,62,63,64], in vascular occlusion/occlusion-like failure studies, in particular [8,9,10,11,12,13,14,15,16,17,18,19,20,21,22,23,24,25] (the counteraction of the increase in malondialdehyde (MDA) values occurred in the present study as well), BPC 157 functions as a stabilizer of cellular junction [6] and mitigates leaky gut syndrome. This is by increasing tight junction protein ZO-1 expression and transepithelial resistance [6], inhibiting the mRNA of inflammatory mediators (iNOS, IL-6, IFN, and TNF-alpha), and increasing the expression of HSP 70 and 90 and antioxidant proteins [6]. Likewise, the inhibition of catabolic pathways (IL-6, TNF-alpha), balanced with the stimulation of anabolic pathways (FoxO3a, p-AKT, p-mTOR, and P-GSK-3β), has occurred in the counteraction of tumor-induced cachexia [56].

Anyway, such BPC 157 therapy might serve as a guided reperfusion recovery [8,9,10,11,12,13,14,15,16,17,18,19,20,21,22,23,24,25]. Effectively upgrading the minor vessel that might take the major function over the function of the failed major vessel that has to be compensated, collaterals to reestablish reorganized blood flow during occlusion/occlusion-like syndrome circumstances, to recover initial ischemia-lesions and attenuate the otherwise inescapable lesions [8,9,10,11,12,13,14,15,16,17,18,19,20,21,22,23,24,25], should be fully operative in full reperfusion conditions as well. This may occur following prolonged intra-abdominal hypertension grade III and grade IV. Given the significance of this effect as a shared effect also in the worst circumstances, BPC 157 therapy (10 µg/kg, 10 ng/kg, given subcutaneously), might serve to counteract these lesions also in the advanced stage and ascertain the counteraction of the existing lesions, instead of further worsening during the subsequent major reperfusion period.

## 2. Results

The post-decompression acute abdominal compartment period characterized reperfusion with the additional vascular failure, and the additional perilous syndrome occurred peripherally and centrally, providing a course that might be not spontaneously recovered. Before the reperfusion and subsequent medication, the rats that barely survived the considerable period of intra-abdominal hypertension grade III and grade IV, the vessel and organ compression, exhibited advanced vascular and multiorgan failure [8]. There was full occlusion/occlusion-like syndrome, additionally presented with intracranial (superior sagittal sinus) hypertension, portal, and caval hypertension, aortal hypotension, progressed venous and arterial thrombosis peripherally and centrally, congested (i.e., inferior caval vein and superior mesenteric vein) and/or failed (i.e., azygos vein) major veins, and ECG disturbances. The brain damage appeared in all investigated areas. In the cerebral and cerebellar cortex, hypothalamus/thalamus, and hippocampus, it appeared as congestion, edema, and a larger area with an increased number of karyopyknotic cells and intracerebral hemorrhage, mostly in the infratentorial space, affecting the cerebello-angle/area. The rats presented myocardial congestion and subendocardial infarction; severe congestion; and hemorrhage in the lung, liver, and kidney. These were all attenuated/eliminated by BPC 157 therapy given during the compression period [8]. In particular, in the gastrointestinal tract, they had considerable lesions, with an increase in severity from the upper to the lower part of the gastrointestinal tract. Likewise, the transmural hyperemia of the gastrointestinal tract, stomach, duodenum, and small and large bowel walls were counteracted, and there was a reduction in the number of villi in the intestinal mucosa and a reduction of the crypt, with the focal denudation of superficial epithelia, as well as a dilatation of the large bowel [8].

On the other hand, in the post-decompression acute abdominal compartment period, the circumstances alternating ischemia with reperfusion are much more demanding for the delayed therapy application during reperfusion. However, the results demonstrated the strong effects of the BPC 157 therapy given during reperfusion. Thus, by rapidly counteracting the reperfusion course, BPC 157 therapy could also reverse previous ischemia-course lesions, thereby inducing complete recovery. Thus, this therapy made this severe vascular and multiorgan failure and occlusion/occlusion-like syndrome, as a whole, fully reversible.

The sustained activation of the azygos vein occurred in all of the BPC 157-treated rats as the common key finding, and was responsible for the prompt effect of the stable gastric pentadecapeptide BPC 157 therapy application. Also, in the advanced reperfusion, the direct blood delivery from the inferior caval vein to the superior caval vein might occur to instantly break the injurious circle. The proof of the concept appeared as the counteraction of the adjacent adverse syndrome (i.e., attenuated/counteracted intracranial (superior sagittal sinus) hypertension and aortal hypotension; major ECG disturbances; progressing arterial and vein thrombosis; and lesions in the brain, heart, lungs, liver, kidneys, and gastrointestinal tract) and counteraction of the increased malondialdehyde (MDA) values in all of the affected organs. While initiated in the worse circumstances of the highly advanced injurious course, in the severely damaged rats, the BPC 157 therapy effect was comparable to the effects of the previous BPC 157 therapy of the mentioned occlusion/occlusion-like syndromes [8,9,10,11,12,13,14,15,16,17,18,19,20,21,22,23,24,25].

### 2.1. A Perilous Syndrome Occurred Peripherally and Centrally

#### 2.1.1. Blood Pressure Disturbances

In previously intra-abdominal hypertensive rats, grade III and grade IV, after decompression and reperfusion initiation, if therapy was not given, the portal and caval hypertension, and even more the intracranial (superior sagittal sinus) hypertension as well as the aortal hypotension persisted and sustainably presented until the end of the experiments. The effectiveness of the therapy application appeared as a prompt reduction in the blood pressure disturbances (superior sagittal sinus, portal, and caval hypertension, and abdominal aorta hypotension) in rats that received BPC 157 therapy. A cause–consequence relation should indicate the beneficial effect occurring both peripherally (with portal and caval hypertension and aortal hypotension almost annihilated) as well as even more centrally (with superior sagittal sinus hypertension attenuated) (Table 1).

#### 2.1.2. Thrombosis

Without BPC 157 therapy, even with the reperfusion, shared thrombosis (in veins and arteries) persisted peripherally, both in veins (i.e., portal vein and inferior caval vein) as well as in arteries (i.e., abdominal aorta) and centrally (i.e., superior sagittal sinus). Likewise, along with reperfusion, BPC 157 therapy might result in a prompt reduction in thrombosis, which appears both peripherally and centrally (Table 1). This might indicate the effective cause–consequence course of the therapy both peripherally and centrally.

#### 2.1.3. Collateral Pathways, Blood Vessels, and Brain Gross Presentation

**Figure 1:**
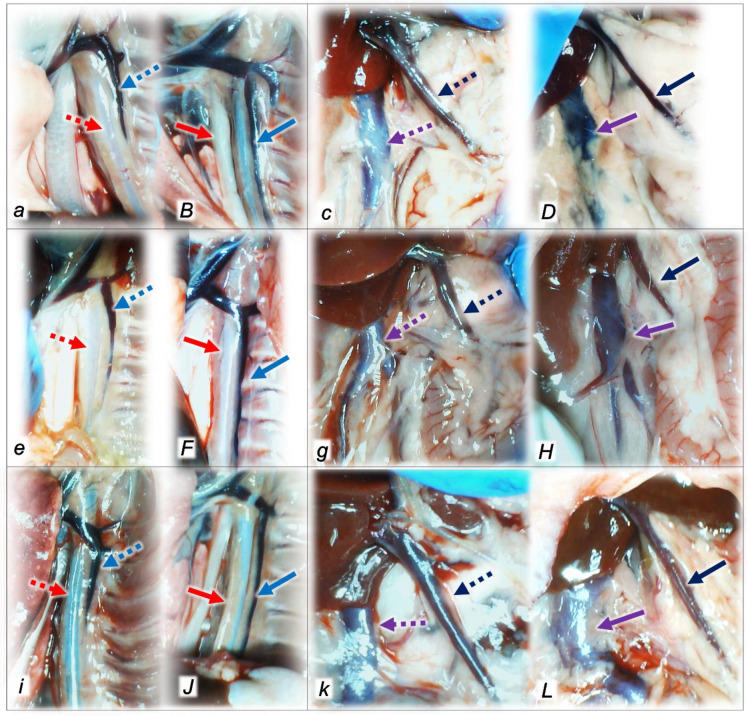
Illustrative presentation of blood vessels (**a**,**B**,**c**,**D**,**e**,**F**,**g**,**H**,**i**,**J**,**k**,**L**) in control rats (small italic letters) (**a**,**c**,**e**,**g**,**i**,**k**) and BPC 157-treated rats (capital italic letters) (**B**,**D**,**F**,**H**,**J**,**L**). Considerable failure or recovery was noted after decompression and reperfusion in rats who were subjected to the intra-abdominal hypertension of 25 mmHg for 60 min (**a**,**B**,**c**,**D**) (i), 30 mmHg for 30 min (**e**,**F**,**g**,**H**) (ii), or 40 mmHg for 30 min (iii) (**i**,**J**,**k**,**L**) and sacrificed after the corresponding reperfusion period (60 min (i) (**a**,**B**,**c**,**D**) or 30 min (ii (**e**,**F**,**g**,**H**); iii (**i**,**J**,**k**,**L**))) depending on whether they had received (sc) saline (controls) or BPC 157 at 3 min reperfusion times. Commonly, controls presented the marked congestion of the superior mesenteric vein (dashed black arrows) and inferior caval vein (dashed violet arrows) and a collapsed azygos vein (dashed blue arrows) and aorta (dashed red arrows). Contrarily, BPC 157-treated rats exhibited a consistent therapy effect and counteracted failed vessel presentation; the superior mesenteric vein (full black arrows) and inferior caval vein (full violet arrows) reversed to normal vessel presentation; the collapsed azygos vein (full blue arrows) and aorta (full red arrows) fully recovered.

**Figure 2:**
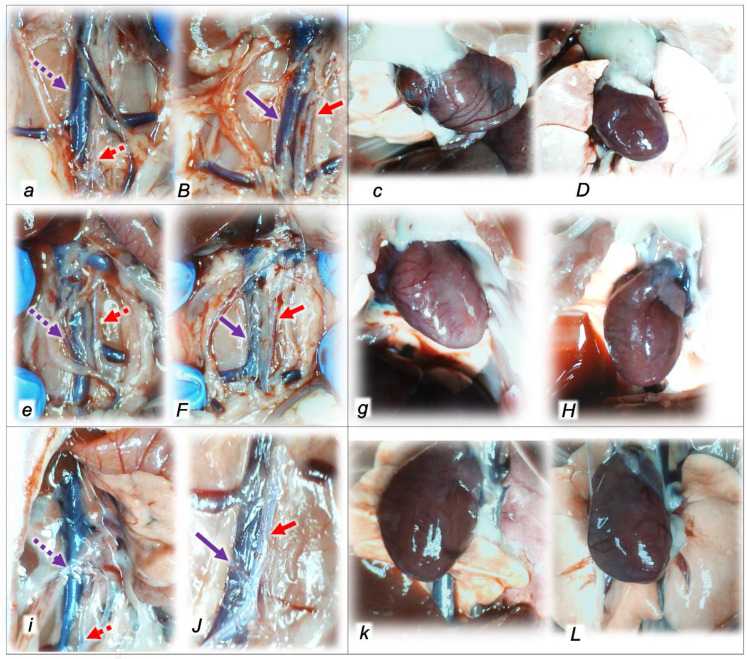
Illustrative presentation of blood vessels and heart (**a**,**B**,**c**,**D**,**e**,**F**,**g**,**H**,**i**,**J**,**k**,**L**) in control rats (small italic letters) (**a**,**c**,**e**,**g**,**i**,**k**) and BPC 157-treated rats (capital italic letters) (**B**,**D**,**F**,**H**,**J**,**L**). Considerable failure or recovery was noted after decompression and reperfusion in rats who were subjected to the intra-abdominal hypertension of 25 mmHg for 60 min (**a**,**B**,**c**,**D**) (i), 30 mmHg for 30 min (**e**,**F**,**g**,**H**) (ii), or 40 mmHg for 30 min (iii) (**i**,**J**,**k**,**L**) and sacrificed after the corresponding reperfusion period (60 min (i) (**a**,**B**,**c**,**D**) or 30 min (ii (**e**,**F**,**g**,**H**); iii (**i**,**J**,**k**,**L**))) depending on whether they had received (sc) saline (controls) or BPC 157 at 3 min reperfusion times. Commonly, controls presented the marked congestion of the inferior caval vein (dashed violet arrows), a collapsed abdominal aorta (dashed red arrows), and a dilated heart. Contrarily, BPC 157-treated rats exhibited a consistent therapy effect and counteracted failed vessel and heart presentation; the inferior caval vein (full violet arrows) reversed to normal vessel presentation; the abdominal aorta (full red arrows) fully recovered and counteracted heart dilatation.

**Figure 3:**
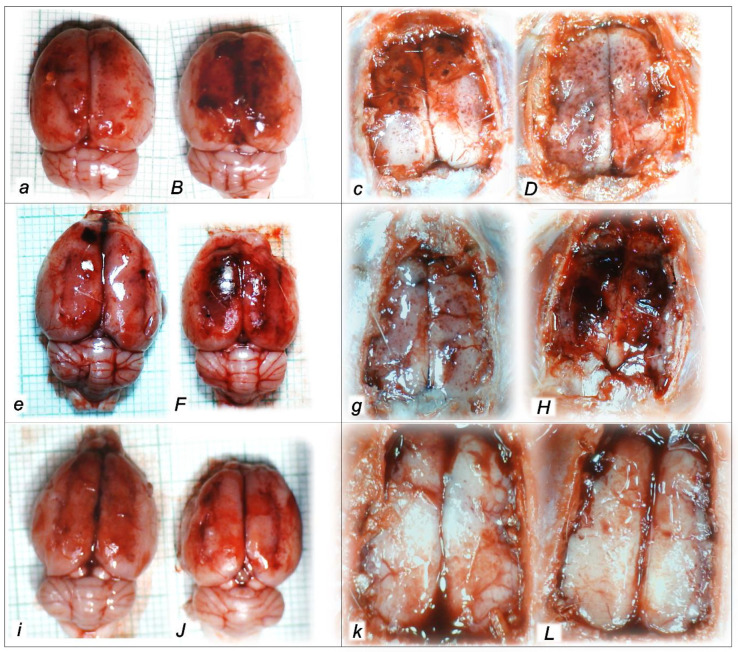
Illustrative brain presentation, ex vivo and in vivo (**a**,**B**,**c**,**D**,**e**,**F**,**g**,**H**,**i**,**J**,**k**,**L**), in control rats (small italic letters) (**a**,**c**,**e**,**g**,**i**,**k**) and BPC 157-treated rats (capital italic letters) (**B**,**D**,**F**,**H**,**J**,**L**). Considerable gross brain failure (swelling) or recovery (counteracted swelling) was noted after decompression and reperfusion in rats who were subjected to the intra-abdominal hypertension of 25 mmHg for 60 min (**a**,**B**,**c**,**D**) (i), 30 mmHg for 30 min (**e**,**F**,**g**,**H**) (ii), or 40 mmHg for 30 min (iii) (**i**,**J**,**k**,**L**) and sacrificed after the corresponding reperfusion period (60 min (i) (**a**,**B**,**c**,**D**) or 30 min (ii (**e**,**F**,**g**,**H**); iii (**i**,**J**,**k**,**L**))) depending on whether they had received (sc) saline (controls) or BPC 157 at 3 min reperfusion times. Commonly, controls presented marked brain swelling. Contrarily, BPC 157-treated rats exhibited a consistent therapy effect and counteracted brain swelling.

Indicatively, for a common clue that might continuously fail during compression and intra-abdominal hypertension and, thereby, also in the subsequent reperfusion after decompression, without therapy, all injured rats without therapy converge to considerable lesions. Thereby, in general (the completed post-intra-abdominal hypertension (grade III and grade IV) course) and in particular (considering each of the given reperfusion time points, (60 min, 30 min, and 30 min)), we might envisage the presentation of the consistently failed collateral pathways as a shared failure (Figure 1, Figure 2 and Figure 3). Contrarily, with BPC 157 therapy, the advanced collateral pathway presentation is initiated immediately upon its delayed application. This might be seen in cause-consequence relation with the reversal of blood pressure disturbances, thrombosis fully counteracted in all vessels investigated, veins and arteries, providing that, peripherally and centrally, the particular vessels recruitment may compensate the major injured vessel failure to take reperfusion blood flow, and counteract blood stasis seeable with, peripherally and centrally. Evidently, relative volume changes (the congested vessels (superior mesenteric vein and inferior caval vein, since the trapped volume, congested liver and lung), dilated heart and collapsed vessels (not functioning azygos vein) and swollen brain) occurred as a part of the previous failure during intra-abdominal hypertension-induced compression and further worsening upon decompression-reperfusion (Table 2). Consequently, the increased relative volume of the superior mesenteric vein and inferior caval vein (congestion) BPC 157 might decrease. The failed volume of the azygos vein (as well as the abdominal aorta) was reversed (i.e., there was an increased relative volume as the azygos vein was reactivated). BPC 157 therapy might bring this vessel and heart presentation close to normal vessel and heart presentation and close to normal functioning to re-establish blood flow (with multiorgan lesion being largely attenuated) (almost annihilated portal and caval hypertension and aortal hypotension). Evidently, in the peripheral–central circuit, at the same time, there was brain swelling, increased intracranial (superior sagittal sinus) hypertension, and an increased volume (associated with considerable brain injuries) (see Section 2.3). BPC 157 administration rapidly counteracted these and induced a considerable decrease toward normal brain presentation and negative pressure values (Table 1).

#### 2.1.4. Heart and ECG Disturbances

We revealed, in the study of the ECG disturbances in the further reperfusion course following the prime acute abdominal compartment and the nodal rhythm, dominant ST elevation, bradycardia, a temporary rescue that occurred following decompression, and a sinus rhythm. Then, since 2 min reperfusion times, there were the nodal rhythm, significant ST elevation, shortened QTc interval, and bradycardia. Extreme bradycardia and asystole appeared as the ultimate outcomes at the end of the investigation period. In BPC 157-treated rats, these disturbances were largely absent during the whole reperfusion period (Table 3). This occurred along with normal heart microscopic presentation, unlike the severe myocardial congestion in controls.

#### 2.1.5. Oxidative Stress

Upon decompression, the increased MDA-value oxidative stress occurred in all investigated tissues given the MDA values (nmol/mg protein) in the brain (6.2 ± 0.2), heart (18 ± 5), lung (15 ± 5), liver (20 ± 6), kidney (10 ± 6), blood (17 ± 5), stomach (1.5 ± 0.3), small intestine (1.1 ± 0.3), and large intestine (0.9 ± 0.3) in the healthy rats. During reperfusion, the highest values occurred in the blood, heart, lung, and kidney, which likely received the greatest impact of the ongoing reperfusion. Lower values appeared in the brain. The lowest values occurred in the gastrointestinal tract. BPC 157 therapy counteracted both the MDA-value increase as well as the oxidative stress damages in all organs tested at the end of all decompression/reperfusion periods, which were investigated to provide additional proof for the concept (Table 4).

### 2.2. A Perilous Syndrome Occurred Peripherally

#### 2.2.1. Gastrointestinal, Lung, Liver, Kidney, and Heart Lesions

**Figure 4:**
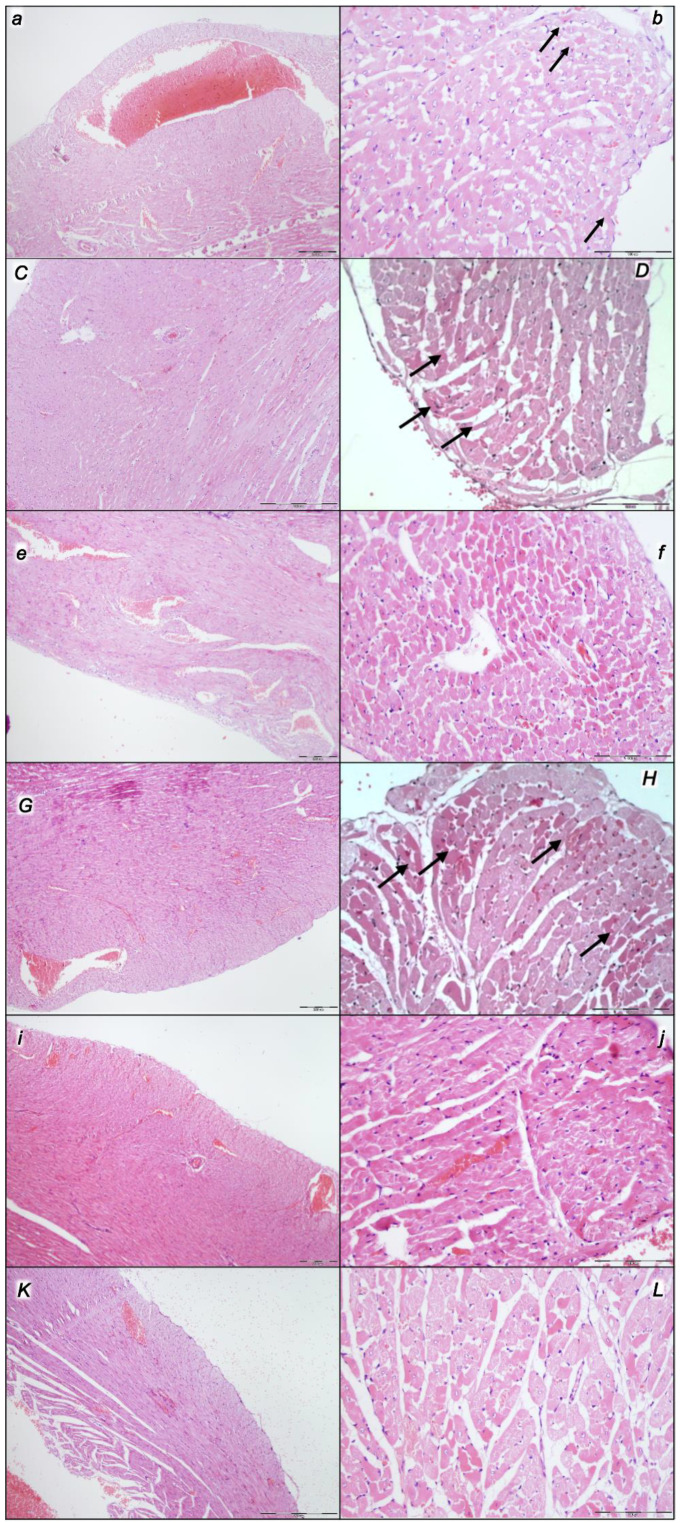
Microscopic changes presented in the heart (**a**,**b**,**C**,**D**,**e**,**f**,**G**,**H**,**i**,**j**,**K**,**L**) in control rats (small italic letters) (**a**,**b**,**e**,**f**,**i**,**j**) and BPC 157-treated rats (capital italic letters) (**C**,**D**,**G**,**H**,**K**,**L**). Considerable lesions were noted after decompression and reperfusion in rats who were subjected to the intra-abdominal hypertension of 25 mmHg for 60 min (**a**,**b**,**C**,**D**) (i), 30 mmHg for 30 min (**e**,**f**,**G**,**H**) (ii), or 40 mmHg for 30 min (iii) (**i**,**j**,**K**,**L**) and sacrificed after the corresponding reperfusion period (60 min (i) (**a**,**b**,**C**,**D**) or 30 min (ii (**e**,**f**,**G**,**H**); iii (**i**,**j**,**K**,**L**)) depending on whether they had received (sc) saline (controls) or BPC 157 at 3 min reperfusion times. Commonly, controls presented the marked congestion of the myocardium with pronounced congestion and the dilatation of coronary arteries and their intramyocardial branches (**a**,**e**,**i**), scattered subendocardial ischemic myocytes (**b**) (black arrows), and subendocardial infarction (**f**,**j**). Contrarily, BPC 157-treated rats exhibited a consistent therapy effect, only mild myocardium congestion (**C**,**G**,**K**), and scattered subendocardial ischemic myocytes (**D**,**H**,**L**) (black arrows). HE staining; magnification, 100×; scale bar, 200 μm (**a**,**C**,**e**,**G**,**i**,**K**) or magnification, 400×; scale bar, 100 μm (**b**,**D**,**f**,**H**,**j**,**L**).

**Figure 5:**
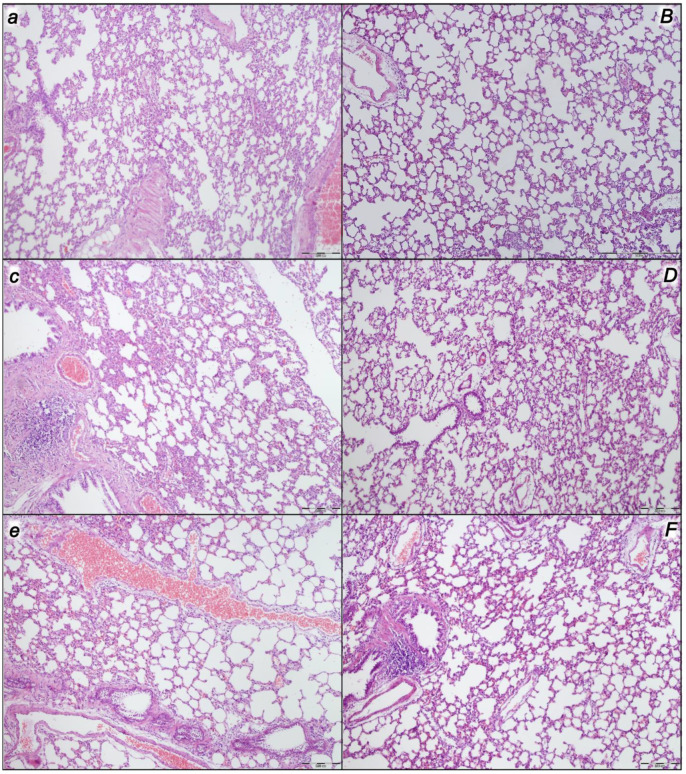
Microscopic changes presented in the lung (**a**,**B**,**c**,**D**,**e**,**F**) in control rats (small italic letters) (**a**,**b**,**e**,**f**,**i**,**j**) and BPC 157-treated rats (capital italic letters) (**B**,**D**,**F**). Considerable lesions were noted after decompression and reperfusion in rats who were subjected to the intra-abdominal hypertension of 25 mmHg for 60 min (**a**,**B**) (i), 30 mmHg for 30 min (**c**,**D**) (ii), or 40 mmHg for 30 min (iii) (**e**,**F**) and sacrificed after the corresponding reperfusion period (60 min (i) (**a**,**B**) or 30 min (ii (**c**,**D**); iii (**e**,**F**)) depending on whether they had received (sc) saline (controls) or BPC 157 at 3 min reperfusion times. Commonly, control animals exhibited the marked congestion of the lung parenchyma, thickening of the alveolar membranes due to capillary congestion, pulmonary edema, and dilatation of larger blood vessels (**a**,**c**,**e**). No changes appeared in the BPC 157-treated rats (**B**,**D**,**F**). HE staining; magnification, 100×; scale bar, 200 μm.

**Figure 6:**
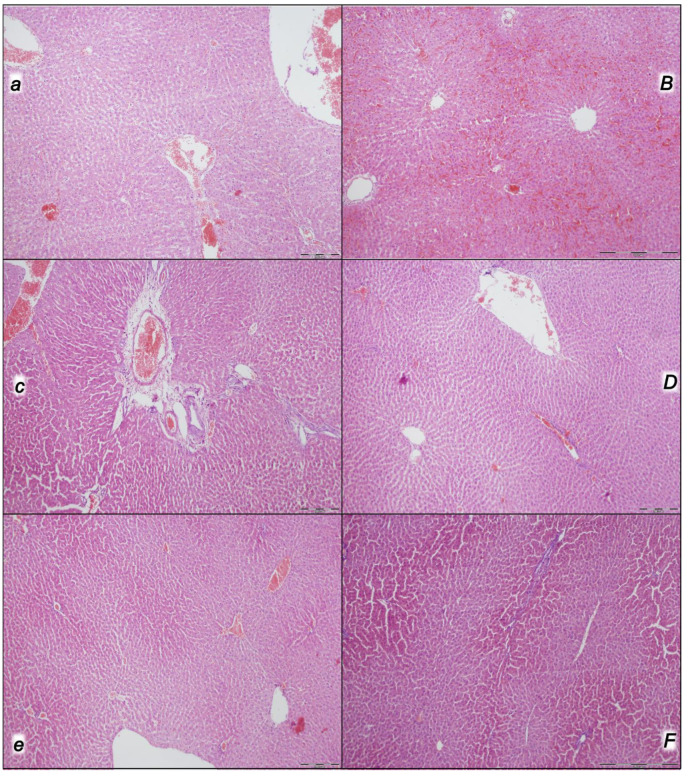
Microscopic changes presented in the liver (**a**,**B**,**c**,**D**,**e**,**F**) in control rats (small italic letters) (**a**,**b**,**e**,**f**,**i**,**j**) and BPC 157-treated rats (capital italic letters) (**B**,**D**,**F**). Considerable lesions were noted after decompression and reperfusion in rats who were subjected to the intra-abdominal hypertension of 25 mmHg for 60 min (**a**,**B**) (i), 30 mmHg for 30 min (**c**,**D**) (ii), or 40 mmHg for 30 min (iii) (**e**,**F**) and sacrificed after the corresponding reperfusion period (60 min (i) (**a**,**B**) or 30 min (ii (**c**,**D**); iii (**e**,**F**)) upon receiving (sc) saline (controls) or BPC 157 at 3 min reperfusion times. Commonly, control animals exhibited the marked congestion of the liver parenchyma, with a pronounced dilatation of sinusoids and branches of the portal vein in portal tracts (**a**,**c**,**e**). No changes or mild congestion appeared in the BPC 157-treated rats (**B**,**D**,**F**). HE staining; magnification, 100×; scale bar, 200 μm.

**Figure 7:**
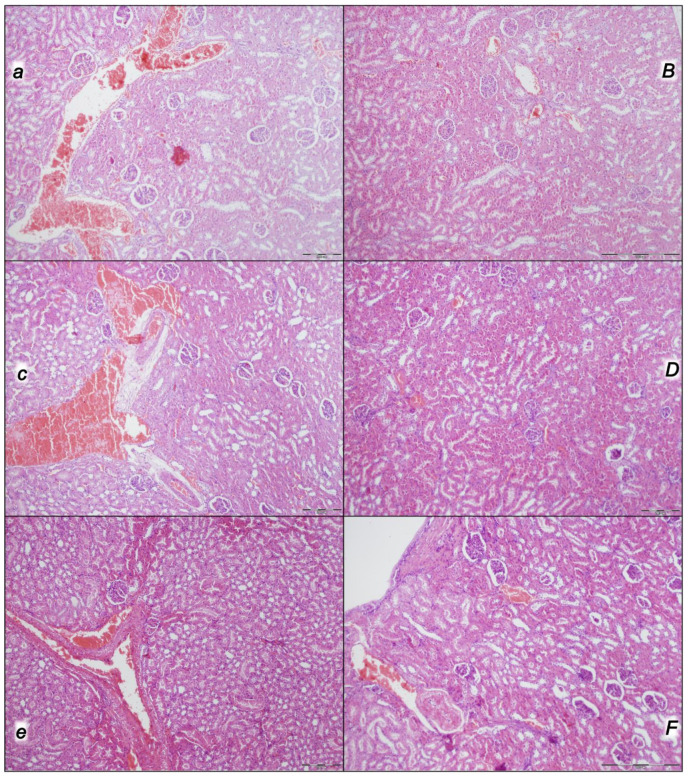
Microscopic changes presented in the kidney (**a**,**B**,**c**,**D**,**e**,**F**) in control rats (small italic letters) (**a**,**b**,**e**,**f**,**i**,**j**) and BPC 157-treated rats (capital italic letters) (**B**,**D**,**F**). Considerable lesions were noted after decompression and reperfusion in rats who were subjected to the intra-abdominal hypertension of 25 mmHg for 60 min (**a**,**B**) (i), 30 mmHg for 30 min (**c**,**D**) (ii), or 40 mmHg for 30 min (iii) (**e**,**F**) and sacrificed after the corresponding reperfusion period (60 min (i) (**a**,**B**) or 30 min (ii (**c**,**D**); iii (**e**,**F**)) upon receiving (sc) saline (controls) or BPC 157 at 3 min reperfusion times. Commonly, control animals exhibited the marked congestion of the renal parenchyma with moderate vascular congestion, and interstitial edema occurred in control rats (**a**,**c**,**e**). No changes or mild congestion appeared in the BPC 157-treated rats (**B**,**D**,**F**). HE staining; magnification, 100×; scale bar, 200 μm.

**Figure 8:**
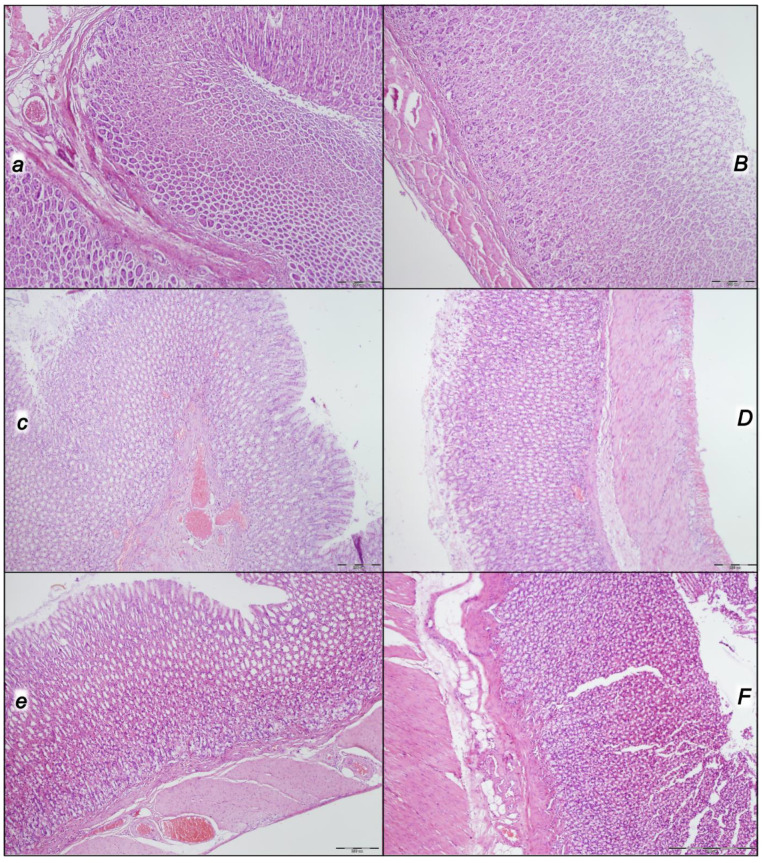
Microscopic changes presented in the stomach (**a**,**B**,**c**,**D**,**e**,**F**) in control rats (small italic letters) (**a**,**b**,**e**,**f**,**i**,**j**) and BPC 157-treated rats (capital italic letters) (**B**,**D**,**F**). Considerable lesions were noted after decompression and reperfusion in rats who were subjected to the intra-abdominal hypertension of 25 mmHg for 60 min (**a**,**B**) (i), 30 mmHg for 30 min (**c**,**D**) (ii), or 40 mmHg for 30 min (iii) (**e**,**F**) and sacrificed after the corresponding reperfusion period (60 min (i) (**a, B**) or 30 min (ii (**c**,**D**); iii (**e**,**F**)) depending on whether they had received (sc) saline (controls) or BPC 157 at 3 min reperfusion times. Commonly, control animals exhibited the marked congestion of the stomach wall due to the transmural pronounced congestion and dilatation of the blood vessels (**a**,**c**,**e**). No changes appeared in the BPC 157-treated rats (**B**,**D**,**F**). HE staining; magnification, 100×; scale bar, 200 μm.

Indicatively, for a failed common clue (i.e., intracranial (superior sagittal sinus), portal, and caval hypertension, and aortal hypotension; progressed thrombosis, peripherally and centrally; failed collateral recruitment; progressed thrombosis) after the regular course following intra-abdominal hypertension-induced compression, there is subsequent decompression and reperfusion confronted with already considerable organ lesions presenting particular post-syndrome (Table 5, Figure 4, Figure 5, Figure 6, Figure 7 and Figure 8).

Contrarily, the reduced severity of lesions due to BPC 157 therapy can be a part of the cause–consequence therapeutic course along with the reduced intracranial (superior sagittal sinus), portal, and caval hypertension, and aortal hypotension; attenuated/eliminated thrombosis; and immediate impact of the activated collateral pathway.

#### 2.2.2. Heart

Given the marked congestion of the myocardium and subendocardial infarcts at the end of the compression period, the initial presentation before decompression and reperfusion might be complex. Thereby, it is interesting that at the end of the reperfusion period, all control rats exhibited pronounced congestion and the dilatation of coronary arteries and their intramyocardial branches; subendocardial infarction occurred in those, which had been subjected to grade IV intra-abdominal hypertension (Table 5, Figure 4). Likewise, as before during the compression period (BPC 157 given at 10 min intra-abdominal hypertension time, the equally high intra-abdominal pressures in BPC 157-treated rats led to preserved myocardium), in the reperfusion there is consistent BPC 157 therapy effect. There was only mild congestion in the myocardium and scattered subendocardial ischemic myocytes in rats who had been subjected to grade III intra-abdominal hypertension, and the same morphology was found in rats who had been subjected to grade IV intra-abdominal hypertension (Table 5, Figure 4). Note that this therapy effect occurred along with the counteracting effect on arrhythmias and thrombosis.

#### 2.2.3. Lung

Without therapy, the initial presentation of marked congestion in the lung at the end of the compression period (i.e., barely survived) appeared at the end of the reperfusion period as a marked congestion of the lung parenchyma, thickening of the alveolar membranes due to capillary congestion, pulmonary edema, and dilatation of larger blood vessels. Contrarily, no changes appeared in the BPC 157-treated rats (Table 5, Figure 5).

#### 2.2.4. Liver

Without therapy, the initial presentation of a marked vascular dilation of the liver parenchyma at the end of the compression period (i.e., barely survived) appeared at the end of the reperfusion period as a pronounced dilatation of sinusoids and branches of the portal vein in portal tracts. Contrarily, BPC 157-treated rats presented with no changes or only mild congestion in the liver (Table 5, Figure 6).

#### 2.2.5. Kidney

Without therapy, the initial presentation of renal congestion at the end of the compression period (i.e., barely survived) course at the end of the reperfusion period appeared as marked congestion of the renal parenchyma with moderate vascular congestion, and interstitial edema. Contrarily, either no changes or only mild congestion appeared in the BPC 157-treated rats (Table 5, Figure 7).

#### 2.2.6. Gastrointestinal Lesion

Without therapy, the initial presentation of marked transmural hyperemia of the entire gastrointestinal tract, stomach, duodenum, and small and large bowel wall at the end of the compression period (i.e., barely survived) remained at the end of the reperfusion period as a marked transmural pronounced congestion and dilatation of the blood vessels (note: small hemorrhagic lesions occurred in the stomach). No changes were found in BPC 157-treated rats (Table 5, Figure 8).

### 2.3. A Perilous Syndrome Occurred Centrally

#### 2.3.1. Brain Lesions, Cerebral and Cerebellar Cortex, Hypothalamus/Thalamus, and Hippocampus

**Figure 9:**
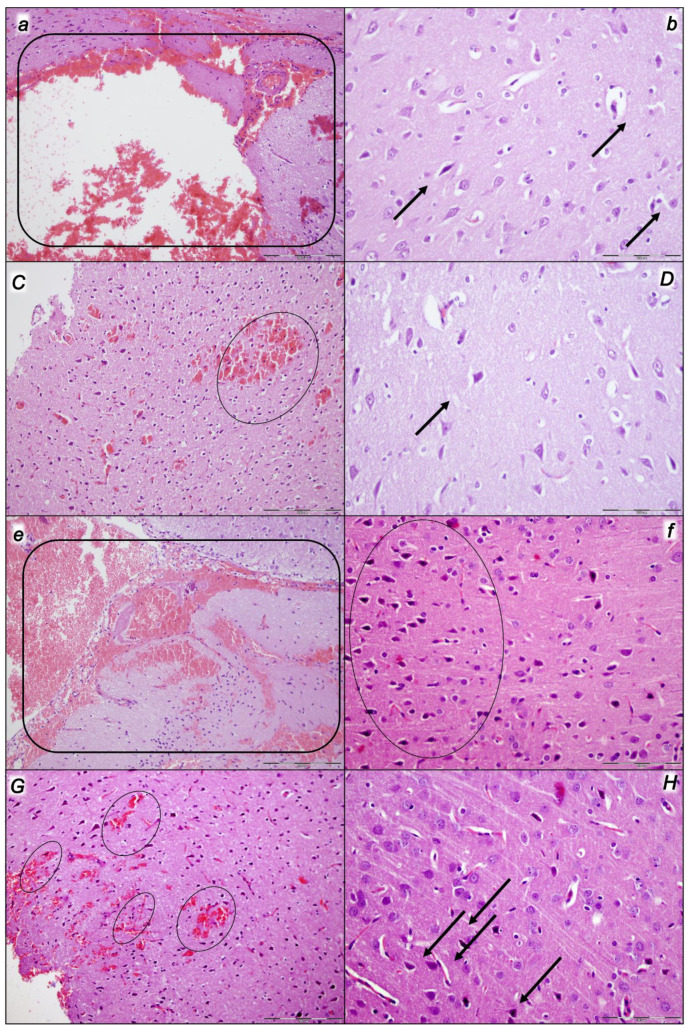
Neuropathological changes presented in the cerebrum (**a**,**b**,**C**,**D**,**e**,**f**,**G**,**H**) in control rats (small italic letters) (**a**,**b**,**e**,**f**) and BPC 157-treated rats (capital italic letters) (**C**,**D**,**G**,**H**). Considerable lesions were noted after decompression and reperfusion in rats who were subjected to the intra-abdominal hypertension of 25 mmHg for 60 min (**a**,**b**,**C**,**D**) (i) or 40 mmHg for 30 min (ii) (**e**,**f**,**G**,**H**) and sacrificed after the corresponding reperfusion period (60 min (i) (**a**,**b**,**C**,**D**) or 30 min (ii)) (**e**,**f**,**G**,**H**) depending on whether they had received (sc) saline (controls) or BPC 157 at 3 min reperfusion times. Commonly, controls presented a pronounced edema and congestion in the brain tissue. In the BPC 157-treated rats (capital italic letters), only mild edema in the brain tissue was found. A focal and deeper neocortical hemorrhage was found in control animals affecting the neocortex, corpus callosum, amygdala, and striatum in the brain tissue (marked areas—(i): (**a**); (ii): (**e**)). In the BPC 157 group, only smaller areas of neocortical hemorrhage occurred (marked areas—(i): (**C**); (ii): (**G**)). Moderate to severe neurodegenerative changes were presented in the cerebral cortex, along with the karyopyknosis of cortical neurons, in controls (marked areas, black arrows) ((i): (**b**); (ii): (**f**)) while BPC 157 rats exhibited, consistently, only rare karyopyknotic cells and mild neurodegenerative changes ((i): (**D**); (ii): (**H**)). HE staining; magnification, 200×; scale bar, 200 μm.

**Figure 10:**
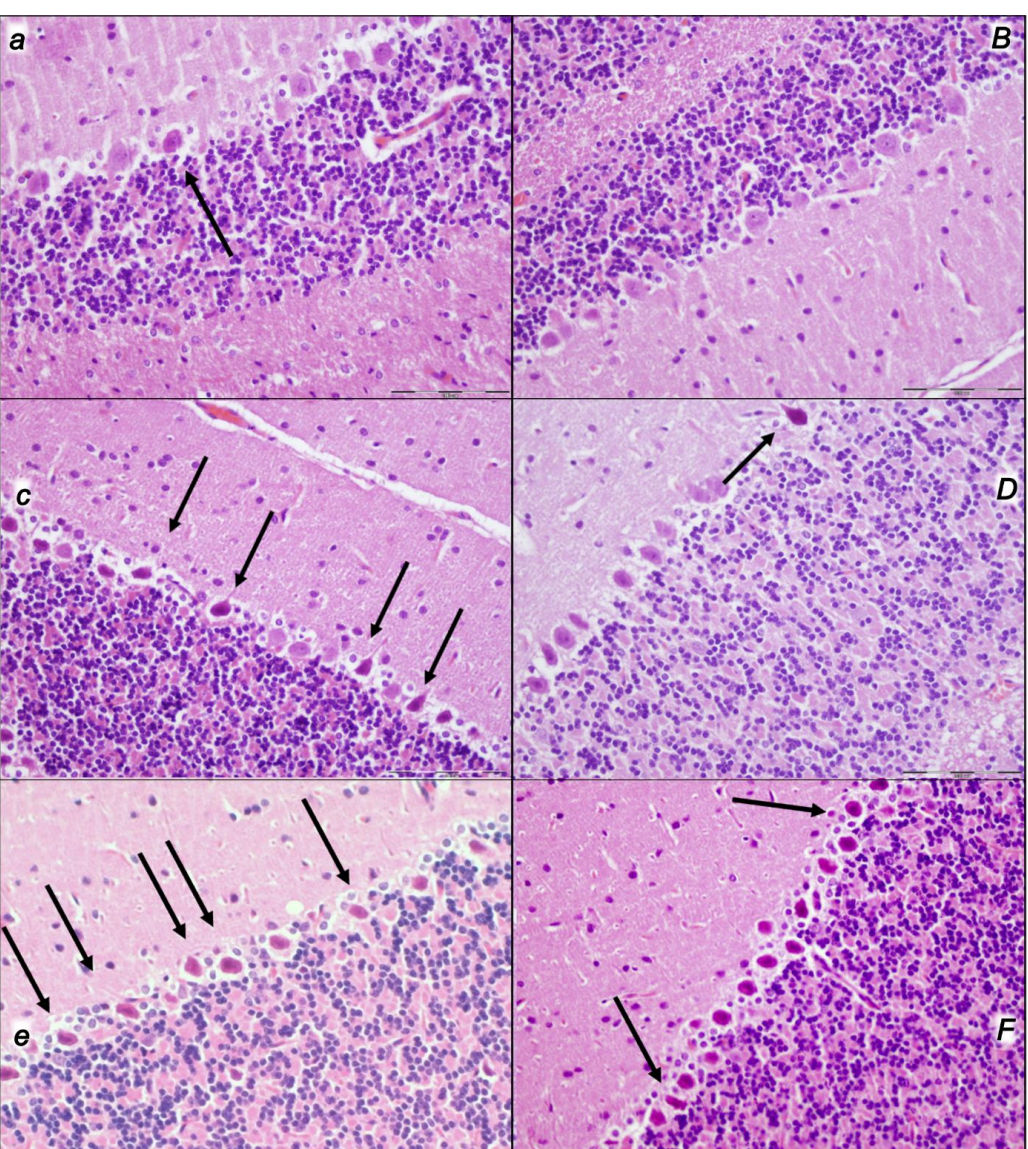
Neuropathological changes presented in the cerebellum (**a**,**B**,**c**,**D**,**e**,**F**) in control rats (small italic letters) (**a**,**c**,**e**) and BPC 157-treated rats (capital italic letters) (**B**,**D**,**F**). Considerable lesions were noted after decompression and reperfusion in rats who were subjected to the intra-abdominal hypertension of 25 mmHg for 60 min (**a**,**B**) (i), 30 mmHg for 30 min (ii) (**c**,**D**), or 40 mmHg for 30 min (iii) (**e**,**F**) and sacrificed after the corresponding reperfusion period (60 min (i) (**a**,**B**) or 30 min ((ii) (**c**,**D**), and (iii) (**e**,**F**))) depending on whether they had received (sc) saline (controls) or BPC 157 at 3 min reperfusion times. Commonly, in the brain tissue, pronounced edema and congestion occurred in controls. Moderate neurodegenerative changes in the cerebellar cortex were found in control animals. There were karyopyknosis and the degeneration of Purkinje cells of the cerebellar cortex (black arrows). In BPC 157 rats, only mild edema in the brain tissue was found, and only rare karyopyknotic cells and mild neurodegenerative changes in the cerebellar cortex occurred (black arrows). HE staining; magnification, 400×; scale bar, 100 μm.

**Figure 11:**
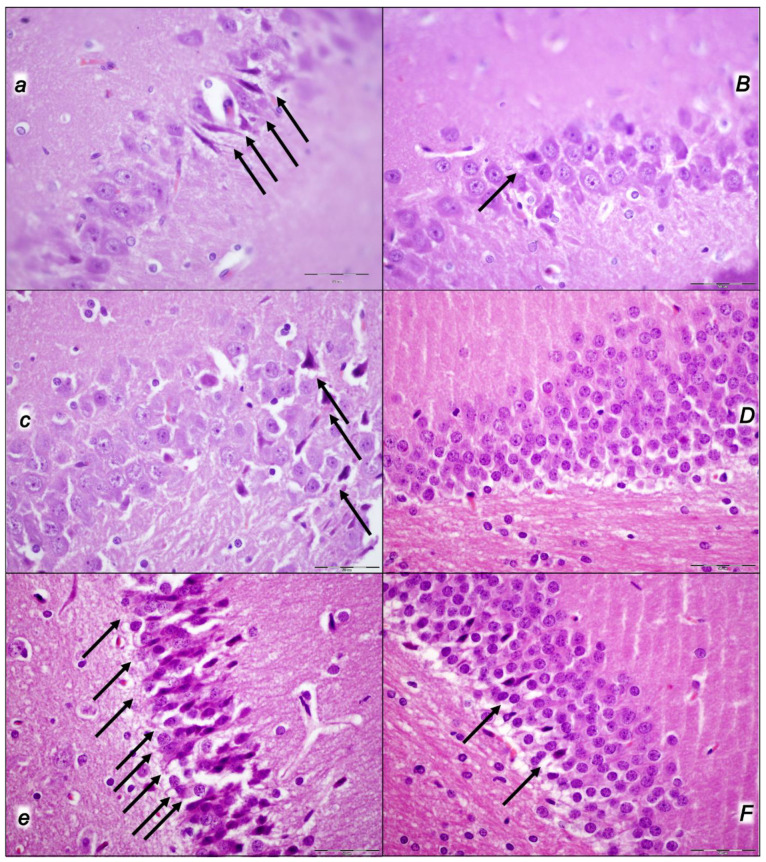
Neuropathological changes presented in the hippocampus (**a**,**B**,**c**,**D**,**e**,**F**) in control rats (small italic letters) (**a**,**c**,**e**) and BPC 157-treated rats (capital italic letters) (**B**,**D**,**F**). Considerable lesions were noted after decompression and reperfusion in rats who were subjected to the intra-abdominal hypertension of 25 mmHg for 60 min (**a**,**B**) (i), 30 mmHg for 30 min (ii) (**c**,**D**), or 40 mmHg for 30 min (iii) (**e**,**F**) and sacrificed after the corresponding reperfusion period (60 min (i) (**a**,**B**), 30 min ((ii) (**c**,**D**) and (iii) (**e**,**F**))) depending on whether they had received (sc) saline (controls) or BPC 157 at 3 min reperfusion times. Commonly, in the brain tissue, pronounced edema and congestion occurred in controls. In the hippocampus, there were moderate neurodegenerative changes in the control animals. There were karyopyknosis and the degeneration of pyramidal cells of the hippocampus (black arrows). BPC 157 rats exhibited only mild edema in the brain tissue and no or only rare karyopyknotic cells in the hippocampus (black arrows). HE staining; magnification, 600×, scale bar, 50 μm.

**Figure 12:**
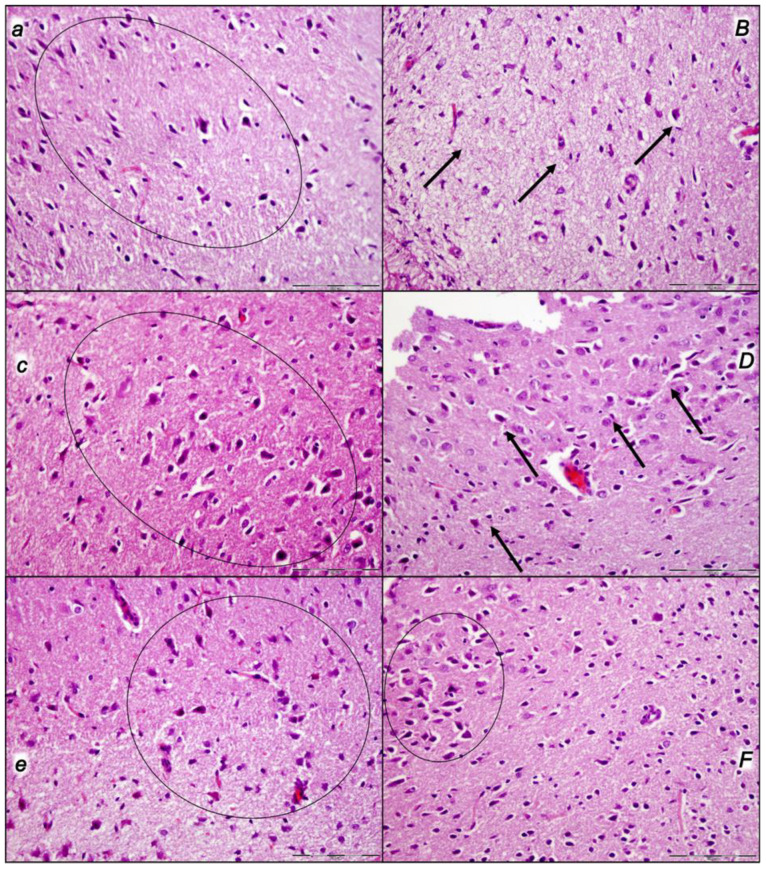
Neuropathological changes presented in the hypothalamus (**a**,**B**,**c**,**D**,**e**,**F**) in control rats (small italic letters) (**a**,**c**,**e**) and BPC 157-treated rats (capital italic letters) (**B**,**D**,**F**). Considerable lesions were noted after decompression and reperfusion in rats who were subjected to the intra-abdominal hypertension of 25 mmHg for 60 min (**a**,**B**) (i), 30 mmHg for 30 min (ii) (**c**,**D**), or 40 mmHg for 30 min (iii) (**e**,**F**) and sacrificed after the corresponding reperfusion period (60 min (i) (**a**,**B**), 30 min ((ii) (**c**,**D**) and (iii) (**e**,**F**))) depending on whether they had received (sc) saline (controls) or BPC 157 at 3 min reperfusion times. Commonly, in the brain tissue, pronounced edema and congestion occurred in controls. In the hippocampus, there were moderate to severe neurodegenerative changes in the control animals. There were karyopyknosis hypothalamic neurons (black arrows). BPC 157 rats exhibited only mild edema in the brain tissue and only rare karyopyknotic cells and mild neurodegenerative changes in the hypothalamus (black arrows). HE staining; magnification, 400×; scale bar, 100 μm.

The applied procedures included intra-abdominal hypertension grade III intra-abdominal hypertension (25 mmHg for 60 min) and grade IV intra-abdominal hypertension (30 mmHg and 40 mmHg both for 30 min) followed by decompression, and reperfusion for corresponding same periods before sacrifice. Thus, equal time-periods of ischemia and reperfusion were involved (60 min/60 min for grade III and 30 min/30 min for grade IV). Thus, the regular severe intra-abdominal hypertension-induced lesions were further affected with decompression and an equal time-period of reperfusion to produce the final considerable ischemia/reperfusion lesions and hemorrhage (i.e., intraventricular hemorrhage involving the third ventricle occurred at the end in rats with grade IV 40 mmHg intra-abdominal hypertension ischemia/reperfusion). In particular, these brain lesions appeared to be distinctively affected by high intra-abdominal pressure and reperfusion; in particular, with higher intra-abdominal hypertension, the cerebrum and hypothalamus were less affected, and the cerebellum and hippocampus were more affected (i.e., the most progressive hippocampal neuronal damage occurred with the highest intra-abdominal pressure). Thereby, after reperfusion, the lesion severity evidence of the major impact on the cerebrum and hypothalamus, and the weaker impact on the cerebellum and hippocampus, appeared as these brain lesions were distinctively affected by reperfusion following high intra-abdominal pressure. All brain lesions were attenuated/eliminated by BPC 157 therapy given in reperfusion (Table 6, Figure 9, Figure 10, Figure 11 and Figure 12).

#### 2.3.2. Cerebrum

Regularly, all of the applied procedures—intra-abdominal hypertension of 25 mmHg for 60 min, 30 mmHg for 30 min, and 40 mmHg for 30 min, followed by decompression and reperfusion for corresponding periods before sacrifice—produced considerable lesions and hemorrhage. These results, however, considerably changed depending on the given medication, i.e., saline (controls) or BPC 157, at 3 min reperfusion times. Commonly, pronounced edema and congestion in the brain tissue were presented in controls. Contrarily, BPC 157-treated rats presented only mild edema in the brain tissue. A focal and deeper neocortical hemorrhage was found in control rats affecting the neocortex, the corpus callosum, the amygdala, and the striatum in the brain tissue. Contrarily, BPC 157 rats presented only smaller areas of neocortical hemorrhage. Likewise, controls exhibited moderate to severe neurodegenerative changes, which were presented in the cerebral cortex, and the karyopyknosis of cortical neurons, while BPC 157 rats exhibited consistently no or only mild neurodegenerative changes (Table 6, Figure 9).

#### 2.3.3. Cerebellum

Commonly, controls presented pronounced edema and congestion in the brain tissue and moderate neurodegenerative changes in the cerebellar cortex. There were karyopyknosis and the degeneration of Purkinje cells of the cerebellar cortex. Contrarily, BPC 157 rats had only mild edema in the brain tissue and only mild neurodegenerative changes in the cerebellar cortex (Table 6, Figure 10).

#### 2.3.4. Hippocampus

Commonly, along with pronounced edema and congestion occurring in the brain tissue, control rats exhibited, in the hippocampus, moderate neurodegenerative changes, karyopyknosis, and the degeneration of pyramidal cells. Contrarily, BPC 157 rats exhibited only mild edema in the brain tissue and had no or only rare karyopyknotic cells in the hippocampus (Table 6, Figure 11).

#### 2.3.5. Hypothalamus

Commonly, along with pronounced edema and congestion in the brain tissue, control rats presented, in the hippocampus, moderate neurodegenerative changes and karyopyknosis hypothalamic neurons. BPC 157 rats exhibited only mild edema in the brain tissue and no or only mild neurodegenerative changes in the hypothalamus (Table 6, Figure 12).

Thus, without given therapy, we evidenced pronounced edema and congestion in the brain tissue and focal and deeper neocortical hemorrhage in all control rats. All four regions—cerebral and cerebellar cortex, hypothalamus/thalamus, and hippocampus—had been affected by neurodegenerative changes. There were karyopyknosis and the degeneration of Purkinje cells of the cerebellar cortex and the karyopyknosis of cortical neurons and pyramidal cells of the hippocampus, as well as hypothalamic neurons. As mentioned before, these were present along with severe gross brain swelling, volume increase, and intracranial (superior sagittal sinus) hypertension. Contrarily, the BPC 157 therapy effect could be summarized as only mild edema and congestion in the brain tissue, with smaller areas of neocortical hemorrhage, visible only within superficial layers of the neocortex, as well as no or only rare karyopyknotic cells in all four regions. Severe gross brain swelling, volume increase, and intracranial (superior sagittal sinus) hypertension had been counteracted.

In summary, rats that had acute abdominal compartments, when undergoing decompression and reperfusion after BPC 157 therapy, exhibited no portal and caval hypertension, ameliorated aortal hypotension, markedly attenuated superior sagittal sinus hypertension, and no disturbed QTc interval. Additionally, venous and arterial thrombosis were almost annihilated, both peripherally and centrally; the lesions were counteracted, and the MDA value increases in the blood, brain, heart, lung, liver, kidney, and gastrointestinal tract were counteracted. Thus, BPC 157 therapy, given in either of the regimens (µg, ng), counteracted the adverse effects that would otherwise consistently appear along with post-acute abdominal compartment syndrome. The key finding regarding a particular activated collateral pathway, i.e., the azygos vein, which combined the inferior caval vein and left superior vein to reorganize blood flow, might be responsible for the noted beneficial effects.

Given the final beneficial result, it seems that as resolving by the BPC 157 therapy works during intra-abdominal hypertension [8], 25 mm Hg for 60 min, 30 mmHg for 30 min, and 40 mmHg for 30 min, it works even more given much later after decompression, during reperfusion and along with reperfusion. These can be two mutually supporting pieces (i.e., activation of the collateral pathways, azygos vein direct blood flow delivery, therapy given at 10 min compression-time, and established severe intra-abdominal hypertension [8]; activation of the collateral pathways, azygos vein direct blood flow delivery, therapy given after decompression, at 3 min reperfusion-time, and already advanced reperfusion) of the consistent vascular recovery evidence. This shows that BPC 157 therapy effectively resolves comparable periods of reperfusion (60 min after 60 min-intra-abdominal hypertension 25 mmHg; 30 min after 30 min-intra-abdominal hypertension 30 mm Hg; and 30 min after 30 min-intra-abdominal hypertension 40 mmHg).

## 3. Discussion

BPC 157 therapy, given in reperfusion, strongly counteracted the decompression/reperfusion-induced occlusion/occlusion-like syndrome that followed the worst circumstances, i.e., occlusion/occlusion-like syndrome of the acute abdominal compartment and intra-abdominal hypertension, grade III and grade IV, in rats, as a whole. This may be indicative and likely for translation, as reperfusion syndrome in patients can frequently occur during decompression surgery and be a cause of death. It was claimed that no therapeutic approach could improve the progression of the syndrome by itself [65,66,67,68]. On the other hand, the cytoprotection background and maintenance and/or recovery of endothelium function [1,2,3,30,31,32,33,34] in favor of this study (reperfusion-induced vascular and multiorgan failure, and occlusion/occlusion-like syndrome as a whole, strongly counteracted by BPC 157 therapy) provided a large beneficial range of effects of BPC 157 therapy that can be likely extended from the previous studies [8,9,10,11,12,13,14,15,16,17,18,19,20,21,22,23,24,25]. Evidence was, among others, via activation of the azygos vein and direct blood flow delivery, recently noted in counteraction of the severe occlusion/occlusion-like syndromes in rats with permanent major vessel occlusion and other similar procedures [8,9,10,11,12,13,14,15,16,17,18,19,20,21,22,23,24,25]. Commonly [8,9,10,11,12,13,14,15,16,17,18,19,20,21,22,23,24,25], based on the beneficial effects consistently noted, it is likely that with the activation of the rescuing collateral pathways (i.e., azygos vein), the venous system has properly been adapted to ascertain proper functioning. Such proper functioning occurs despite permanent vessel occlusion, or other applied noxious procedures, centrally (i.e., with intracranial (superior sagittal sinus) hypertension attenuated) and peripherally (i.e., with portal and caval hypertension eliminated and aortal hypotension attenuated). As a part of such a “bypassing key”, the lesions and hemorrhage in the brain, heart, lung, liver, kidney, and gastrointestinal tract were counteracted, thrombosis in veins and arteries (and stasis) was almost annihilated, and advanced Virchow triad circumstances were fully reversed [8,9,10,11,12,13,14,15,16,17,18,19,20,21,22,23,24,25]. In particular, for acute abdominal compartment syndrome, even the most affected rats, with even more severe occlusion/occlusion-like syndrome, resolved ischemia in such a way that they allowed unchanged severe intra-abdominal hypertension grade III and grade IV (i.e., controlled intraperitoneal insufflation of ordinary air), which they smoothly sustained without major harm after receiving BPC 157 therapy [8]. Thereby, as we have evidenced in the present report, this counteracting potential would include excessive reperfusion conditions after prompt decompression. With conceptual notification that vascular rescue rapidly occurs in cytoprotection [36], the significance appears consistently [8,9,10,11,12,13,14,15,16,17,18,19,20,21,22,23,24,25]. This would be prompt counteraction with rapid vessel recovery, ensuring the prompt capability of the vessels to take full blood flow and avoid (or stop) an otherwise imminent course (increased permeability, massive hemorrhage leading hypotension, cardiac failure, and arrhythmias) resulting in high mortality in patients [65,66,67,68].

Together, this evidence has forced focus on the delayed BPC 157 therapy and the beneficial effects noted in those rats in the post-decompression time. Conceptually, reperfusion after decompression in rats that had intra-abdominal hypertension and occlusion/occlusion-like syndrome resolved the implementation [8,9,10,11,12,13,14,15,16,17,18,19,20,21,22,23,24,25] of Robert’s long-standing concept of cytoprotection positing that the cells afforded by a cytoprotection agent should equally resist noxious events [35], whether ischemia or reperfusion, and thereby stands BPC 157 therapy as a particular cytoprotective agent’s effect [1,2,3,30,31,32,33,34]. Likewise, all the lesions may share the failed cytoprotection (vascular failure), and vice versa; in general terms, the endothelium recovery of cytoprotection via cytoprotection agent therapy (i.e., activated azygos vein pathway, direct blood flow delivery) may impact all these lesions as well—a point clearly shown in practice with the pleiotropic beneficial effects of BPC 157 therapy [1,2,3,30,31,32,33,34]. In addition, BPC 157 therapy may corroborate the spontaneous response to counteract the late harmful consequence and exert full recovery. Namely, it may corroborate temporary, short-lasting relief following decompression and reperfusion (i.e., sinus rhythm (while worsening occurs soon thereafter)). In addition, thrombosis and intra-cranial, portal, and caval hypertension after reperfusion were lower than after the compression period. Direct proof may be the rapid change in the lipid contents and protein secondary structure conformation in the vessel wall produced instantly by BPC 157 therapy [59] (Fourier transform infrared spectroscopy) that avoids otherwise imminent cell death and thereby ascertains vessel functioning even in the worst circumstances.

Thus, along with the previous findings [8,9,10,11,12,13,14,15,16,17,18,19,20,21,22,23,24,25], the provided evidence may verify that BPC 157 therapy can be effective when given throughout reperfusion after decompression in rats that have intra-abdominal hypertension and occlusion/occlusion-like syndrome for a considerable period, and that it can counteract the ongoing occlusion/occlusion-like syndrome as a whole and reperfusion-induced occlusion/occlusion-like syndrome in particular. Such a demonstration combines, in therapy terms, the opposite points: the reperfusion after the prime acute abdominal compartment, the dangerous course of occlusion/occlusion-like syndrome, and the post-decompression period and antecedent mechanical compression ischemia severe intra-abdominal hypertension grade III and grade IV occlusion/occlusion-like syndrome. This has occurred in a particular way, given that both were equally counteracted by the successful outcome of BPC 157 therapy. Moreover, to do this, this has revealed that BPC 157, through its rapid vascular effect along with reperfusion, simultaneously also resolved the antecedent ischemia/compression lesions and, thus, the previously mentioned ischemic occlusion/occlusion-like syndrome and further reperfusion occlusion/occlusion-like syndrome.

It is likely that BPC 157 therapy might provide a particular central/peripheral equation during reperfusion [30,31,32,33,34]. There were, as proofs of the concept (the intracranial (superior sagittal sinus), portal, and caval hypertension, and aortal hypotension were counteracted) in either case, a rapid effect, reduced gross (i.e., brain swelling was rapidly counteracted) and microscopic organs lesions, congestion, and hemorrhage. Namely, BPC 157 therapy instantly recovered the heart (severe ECG disturbances and thrombosis were antagonized) as a whole, and also recovered the brain (along with brain swelling gross counteraction, microscopically, the lesions in all brain areas were antagonized). Thereby, simultaneously, an otherwise persisting harmful inability to drain venous blood adequately for a given cerebral blood inflow without raising venous pressures was counteracted, and on the other side, there were counteractions of congestion, hemorrhage, and lesions in the heart, lungs, liver, kidney, and gastrointestinal tract. As the prime or final result, the previously collapsed azygos vein was made fully functional (direct blood flow delivery, activated rescuing pathway), and the previously congested inferior caval and superior mesenteric veins were reversed, via BPC 157 therapy, to the normal vessel presentation. Together, the almost-eliminated thrombosis (peripherally and centrally) in arteries and veins can prove the eliminated general stasis, reorganized blood flow, and eliminated arrhythmias. Note that as before, in the counteraction of amphetamine arrhythmias [24], the shortened QT interval was counteracted. Regularly, this occurs with increased levels of catecholamine (likely to occur in reperfusion), potentially leading to sudden cardiac death [69,70,71,72,73,74]. Further, along with annihilated thrombosis (peripherally and centrally), heart failure recovered as a whole [27,28], and this always appeared as a common effect. Thus, in addition to other occlusion/occlusion-like syndromes [8,9,10,11,12,13,14,15,16,17,18,19,20,21,22,23,24,25], such effectiveness provides a network of therapy evidence in full reperfusion conditions, with heart, brain, and other organ lesions counteracted, thrombosis annihilated, and hemorrhage counteracted, with all these changes supporting each other. Consequently, Virchow triad circumstance reversal goes against one or more targets, each with a particular effect and a specificity depending on the injury, all orchestrated to achieve a “bypassing key” that might reverse the complete multiorgan failure syndrome and occlusion/occlusion-like syndrome, as a whole, in reperfusion conditions [8,9,10,11,12,13,14,15,16,17,18,19,20,21,22,23,24,25].

Moreover, as perceived in all these findings in the original cytoprotective terms (i.e., endothelium protection → epithelium protection, through activated collateral pathways, i.e., azygos vein direct blood flow delivery), essential support could be mentioned for the interaction with the systems essentially involved in cytoprotection and vessel functioning, and for the NO-and-prostaglandins system as a whole [40,41,42,43,44,45,48,49]. The BPC 157 beneficial action included inducing the NO release of its own [43,44] and the counteraction of the adverse effects of either NOS blockade or NOS over-stimulation [40,41,42,43,44,45,48,49]—in particular, the counteraction of L-NAME-hypertension [43] and the pro-thrombotic effect [45] and L-arginine-hypotension [43] and the anti-thrombotic effect [45]. Likewise, there were counteractions of the large range of adverse effects of NSAIDs, both non-specific and specific; COX-1 and COX-2 inhibitors; and COX-2 inhibitors (i.e., ulceration, liver and brain injuries, and bleeding) [45,46,47,75,76,77,78,79]. It is likely that these counteractions might also have occurred due to the particular effect on thrombocyte function maintenance without affecting coagulation pathways [45,46,47], particularly controlling vasomotor tone through the activation of the Src-Caveolin-1-eNOS pathway [48,49]). In addition, the counteraction of the increased malondialdehyde (MDA) values in all organs investigated, i.e., the blood, brain, heart, liver, kidney, and gastrointestinal tract, is the next piece of evidence that BPC 157 therapy exerts its pleiotropic beneficial effects in all rats that have intra-abdominal hypertension (grade III and grade IV), decompression, and reperfusion in a particular way. This might occur as part of the damaged vascular wall (note: the highest MDA values appeared in the blood) and in other cells of the tissues, especially in combination with the oxygen intermediates and defective endothelial production, as emphasized in acute abdominal compartment rescuing pitfalls [80] that would be hardly resolved. Thus, as we have suggested before [6,56,60,61,62,63,64], it may be that BPC 157 therapy accordingly acts as a natural free scavenger, particularly in occlusion/occlusion-like syndrome [8,9,10,11,12,13,14,15,16,17,18,19,20,21,22,23,24,25], counteracting, in reperfusion, the organ lesions as the oxidative stress damages and the consistent counteraction appear whatever the extent of the oxidative stress may be. Thereby, the heart, lung, liver, and kidney sustain major impacts and have high MDA values that affect these organ lesions and vice versa and, once counteracted, indicate the value of the therapy effect. Likewise, the lesions in the brain and gastrointestinal tract with the lower MDA values may be indicative of particular organ sensitivity (i.e., intracerebral hemorrhage), and, vice versa, of particular therapy effect value. As mentioned, these might constitute the BPC 157 selective action in decompression/reperfusion, related to its modulatory effects, at least, on two systems essentially involved in the cytoprotection concept and vascular integrity maintenance, prostaglandin system [40] and NO system [41,42,43,44,45,48,49], and its additional particular interaction with several molecular pathways [6,48,49,50,51,52,53,54,55,56,57,58]. Moreover, this may be a fully controlled response providing, for instance, in the counteraction of tumor-induced cachexia, the inhibition of catabolic pathways (IL-6, TNF-alpha) balanced with the stimulation of anabolic pathways (FoxO3a, p-AKT, p-mTOR, and P-GSK-3β) [6]. This has been associated with its function as a stabilizer of cellular junction [56], leading to significantly mitigated leaky gut syndrome (by increasing tight junction protein ZO-1 expression) and transepithelial resistance [56]. Likewise, there are inhibited the mRNA of inflammatory mediators (iNOS, IL-6, IFN, and TNF-alpha) while increased expression of HSP 70 and 90, and antioxidant proteins, such as HO-1, NQO-1, glutathione reductase, glutathione peroxidase 2, and GST-pi [56]. Note that such counteraction via BPC 157 therapy may be important as blood–brain barrier injury, increased capillary permeability in the brain, increased extracellular fluid, cerebral white matter edema, and, finally, increased intracranial pressure during intra-abdominal hypertension have all been postulated to relate to the release of these cytokines [81,82,83].

Finally, with decompression/reperfusion-occlusion/occlusion-like syndrome (like with other occlusion/occlusion-like syndromes [8,9,10,11,12,13,14,15,16,17,18,19,20,21,22,23,24,25]), a closely interrelated lesion course supports mutual development and therapy. With BPC 157 therapy, there is extended effectiveness. This has been fully demonstrated with multiple targets. Note that initially, these targets were simultaneously affected by intra-abdominal hypertension grade III and grade IV. Subsequently, they were the more damaged targets and were more affected by extended reperfusion. In conclusion, presenting the decompression/reperfusion period as one of severe vascular and multiorgan failure, and particularly decompression/reperfusion -occlusion/occlusion like-syndrome, BPC 157 therapy exerts a particular action to organize the bypassing of the defect, blood flow re-establishing, and reorganization to compensate vascular defects and/or reverse induced failure [8,9,10,11,12,13,14,15,16,17,18,19,20,21,22,23,24,25]. Consistently, it might be rapidly operative and provide resolution in threatening conditions following decompression and reperfusion.

## 4. Materials and Methods

### 4.1. Animals

This study was conducted with 12 week old, 200 g body weight, male albino Wistar rats, randomly assigned at 6 rats/group/interval. Rats were bred in-house at the Pharmacology Animal Facility, School of Medicine, Zagreb, Croatia. The animal facility was registered by the Directorate of Veterinary (Reg. No: HR-POK-007). Laboratory rats were acclimated for five days and randomly assigned to their respective treatment groups. Laboratory animals were housed in polycarbonate (PC) cages under conventional laboratory conditions at 20–24 °C, relative humidity of 40–70% and noise level 60 dB. Each cage was identified with dates, number of study, group, dose, and number and sex of each animal. Fluorescent lighting provided illumination 12 h per day. Standard good laboratory practice (GLP) diet and fresh water were provided ad libitum. Animal care was in compliance with standard operating procedures (SOPs) of the Pharmacology Animal Facility and the European Convention for the Protection of Vertebrate Animals used for Experimental and other Scientific Purposes (ETS 123). This study was approved by the local Ethics Committee. Ethical principles of the study complied with the European Directive 010/63/E, the Law on Amendments to the Animal Protection Act (Official Gazette 37/13), the Animal Protection Act (Official Gazette 135/06), the Ordinance on the protection of animals used for scientific purposes (Official Gazette 55/13), Federation of European Laboratory Animal Science Associations (FELASA) recommendations, and the recommendations of the Ethics Committee of the School of Medicine, University of Zagreb. The experiments were assessed by observers blinded to the treatment.

### 4.2. Drugs

Medication was administered as described previously [8,9,10,11,12,13,14,15,16,17,18,19,20,21,22,23,24], without the use of a carrier or peptidase inhibitor, for stable gastric pentadecapeptide BPC 157, a partial sequence of the human gastric juice protein BPC, which is freely soluble in water at pH 7.0 and in saline. BPC 157 (GEPPPGKPADDAGLV, molecular weight 1419; Diagen, Slovenia) was prepared as a peptide with 99% high-performance liquid chromatography (HPLC) purity, with 1-des-Gly peptide being the main impurity. The dose and application regimens were as described previously [8,9,10,11,12,13,14,15,16,17,18,19,20,21,22,23,24,25].

### 4.3. Experimental Protocol

In deeply anesthetized rats (intraperitoneal (ip) injection of 40 mg/kg thiopental (Rotexmedica, Trittau, Germany) and 10 mg/kg diazepam (Apaurin; Krka, Novo Mesto, Slovenia)), we induced abdominal compartment syndrome with intraabdominal hypertension grade III and grade IV as described before [8]. Intraperitoneal insufflation of ordinary air was controlled using a manual and digital manometer with a data lodger connected to a computer (DD890, Dostmann Electronic GmbH, Wertheim, Germany). Increased abdominal pressure was maintained (measurement interval of 1 s) at 25 mmHg for 60 min, at 30 mm for 30 min, and at 40 mm Hg for 30 min until the decompression (laparotomy and calvariectomy) and then followed by reperfusion for 60 min (25 mmHg), 30 min (30 mm Hg), or 30 min (40 mm Hg) until sacrifice. Rats received (/kg sc) BPC 157 (10 µg, 10 ng) or saline (5 mL) at 3 min reperfusion time.

Recording of the brain swelling was performed in rats before sacrifice. Briefly, for calvariectomy, 6 burr holes were drilled in three horizontal lines, all of them medially to the superior temporal lines and temporalis muscle attachments. The rostral two burr holes were placed just basally from the posterior interocular line, the basal two burr holes were placed just rostral to the lambdoid suture (and transverse sinuses) on both sides, and the middle two burr holes were placed in the line between the basal and rostral burr holes.

Laparatomized rats were checked for the corresponding presentation of the peripheral vessels (azygos vein, superior mesenteric vein, portal vein, inferior caval and abdominal aorta). Recording with a camera attached to a VMS-004 Discovery Deluxe USB microscope (Veho, Claymont, DE, USA) was performed until the end of the experiment and assessed at 60 min (25 mmHg), 30 min (30 mm Hg), or 25 min (40 mm Hg) reperfusion time.

### 4.4. Superior Sagittal Sinus, Portal, Superior Mesenteric and Caval Vein and Abdominal Aorta: Pressure Recording

As described before [8,9,10,11,12,13,14,15,16,17,18,19,20,21,22,23,24,25], recordings were made in deeply anesthetized rats after with a cannula (BD Neoflon™ Cannula) connected to a pressure transducer (78534C MONITOR/TERMINAL; Hewlett Packard, Palo Alto, CA, USA), which was inserted into the portal vein, inferior caval vein and superior sagittal sinus, and abdominal aorta at the level of the bifurcation at 60 min (25 mmHg), 30 min (30 mm Hg), or 25 min (40 mm Hg) reperfusion time. The superior sagittal sinus anterior part was cannulated by Braun intravenous cannulas, and then, the portal vein, inferior vena cava, and abdominal aorta were cannulated for pressure recording.

Notably, normal rats exhibited a superior sagittal sinus pressure of −24–−27 mmHg and superior mesenteric pressure and portal pressure of 3–5 mmHg, which were similar to those of the inferior vena cava, though with at least 1 mmHg higher values in the portal vein. In contrast, abdominal aorta blood pressure values were 100–120 mm Hg at the level of the bifurcation [8,9,10,11,12,13,14,15,16,17,18,19,20,21,22,23,24,25].

### 4.5. ECG Recording

ECGs were recorded continuously in deeply anesthetized rats for all three main leads by positioning stainless steel electrodes on all four limbs using an ECG monitor with a 2090 programmer (Medtronic, USA) connected to a Waverunner LT342 digital oscilloscope (LeCroy, Chestnut Ridge, NY, USA) until the 60 min (25 mmHg), 30 min (30 mm Hg), or 30 min (40 mm Hg) reperfusion time. This arrangement enabled precise recordings, measurements, and analysis of ECG parameters [8,9,10,11,12,13,14,15,16,17,18,19,20,21,22,23,24,25].

### 4.6. Thrombus Assessment

On being euthanized, the superior sagittal sinus and, peripherally, portal vein, inferior caval vein, and abdominal aorta were removed from the rats, and clots were weighed [8,9,10,11,12,13,14,15,16,17,18,19,20,21,22,23,24,25].

### 4.7. Brain Volume, Heart Volume, and Vessel Volume Presentation

We applied the procedure used before in our previous vascular studies [8,9,10,11,12,13,14,15,16,17,18,19,20,21,22,23,24,25]. Brain volume, vessel volume, and heart volume were proportional to the changes in the brain, vessel, or heart surface areas. The presentation of the brain and peripheral vessels (superior mesenteric vein, inferior caval vein, azygos vein, and abdominal aorta) was recorded in deeply anaesthetized rats with a camera attached to a VMS-004 Discovery Deluxe USB microscope (Veho, Claymont, DE, USA) [14,15,16,17,18,19,20,21,26,27,28]. The border of the brain (or vessels or heart) in each image was marked using ImageJ software, and then, the surface area of the brain (or veins or heart) was measured. This was done with brain (or vein or heart) images for healthy rats and then for both the control (saline) group and treated (BPC 157) group of rats at same intervals after the application and at the time of sacrifice. The arithmetic mean of the surface areas was calculated for both groups. Then, the ratio of these two areas was calculated as ($\frac{A_{con}}{A_{bpc}}$), where Acon was the arithmetic mean brain (or vein or heart) area of the control group and Abpc was the arithmetic mean brain (or vein or heart) area of the treated group. Starting from the square-cube law in Equations (1) and (2), an equation for the change in brain (or vein or heart) volume proportional to the change in brain (or vein or heart) surface area [6] was derived. In expressions (1–5), *l* is defined as any arbitrary one-dimensional length of the brain (for example, rostro-caudal length of the brain), used only for defining the one-dimensional proportion (l2/l1) between two observed brains (or veins or heart) and as an inter-factor (and because of that, it is not measured [6]) for deriving final expression (6). The procedure was as follows: $A_{2}=A_{1}\times {\left(\frac{l_{2}}{l_{1}}\right)}^{2}$ [1] (square-cube law), $V_{2}=V_{1}\times {\left(\frac{l_{2}}{l_{1}}\right)}^{3}$ [2] (square-cube law), $\frac{A_{2}}{A_{1}}={\left(\frac{l_{2}}{l_{1}}\right)}^{2}$ [3] (from [1], after dividing both sides by A1), $\frac{l_{2}}{l_{1}}=\sqrt{\frac{A_{2}}{A_{1}}}$ [4] (from [3], after taking the square root of both sides), $\frac{V_{2}}{V_{1}}={\left(\frac{l_{2}}{l_{1}}\right)}^{3}$ [5] (from [2], after dividing both sides by V1), and $\frac{V_{2}}{V_{1}}={\left(\sqrt{\frac{A_{2}}{A_{1}}}\right)}^{3}$ [6] (after incorporating expression (4) into Equation (5)).

### 4.8. Gross Assessment of Gastrointestinal Lesions

For recording, we used a camera attached to a VMS-004 Discovery Deluxe USB microscope (Veho, Claymont, DE, USA). As described before, gross lesions in the gastrointestinal tract and in the stomach (sum of the longest diameters, in mm) were assessed in deeply anesthetized rats that were laparatomized before sacrifice [8,9,10,11,12,13,14,15,16,17,18,19,20,21,22,23,24,25].

### 4.9. Microscopy

As described in the previous studies [8,9,10,11,12,13,14,15,16,17,18,19,20,21,22,23,24,25], evaluation was performed using light microscopy using an Olympus 71 digital camera and an Olympus BX51 microscope (OLYMPUS Europa SE & CO. KG, Hamburg, Germany). Digital images were saved as uncompressed 24-bit RGB TIFF files using the software program AnalySIS (Olympus Soft Imaging System GmbH, Munster, Germany). Representative tissue specimens (i.e., of the brain, liver, kidney, stomach, small and large intestine, lungs, and heart, taken at the end of the experiment and fixed in 10% neutral buffered formalin (pH 7.4) at room temperature for 24 h) were embedded in paraffin, sectioned at 4 μm, and stained with hemalaun and eosin (H&E).

#### 4.9.1. Brain Histology 

As described in the previous studies [8,9,10,11,12,13,14,15,16,17,18,19,20,21,22,23,24,25], the brain was dissected according to NTP-7 at Level 3 and 6 with neuroanatomic subsites presented in certain brain sections using coronal sections with three mandatory sections. We used a semiquantitative neuropathological scoring system and sum of analyzed affected areas (0–4) (i) and karyopyknotic cells in the brain areas (0–4) (ii), making (i) + (ii) a combined score (0–8) as follows. (i) Specific affected brain areas (0–4) (score 0 indicates no histopathologic change)—cerebral areas (NTP-7, Level 3), cerebellar cortex (NTP-7, Level 6), hippocampus, thalamus, and hypothalamus (NTP-7, Level 3)—presented as follows: small, patchy, complete or incomplete infarcts (≤10% of area affected)(score 1); partly confluent or incomplete infarcts (20–30% of area affected) (score 2); and large confluent complete infarcts (40–60% of area affected) (score 3). In cortex, there was total disintegration of the tissue; in hypothalamus, thalamus, and hippocampus, there were large complete infarcts (˃75% of area affected) (score 4). (ii) We analyzed karyopyknotic cells in the affected brain areas (0–4) (score 0 indicates no change)—cerebral areas (NTP-7, Level 3), cerebellar cortex (NTP-7, Level 6), hippocampus, thalamus, and hypothalamus (NTP-7, Level 3)—and noted results as follows: a few karyopyknotic neuronal cells (≤20%) (score 1); patchy areas of karyopyknotic cells (50%) (score 2); more extensive karyopyknotic areas (75%) (score 3); and complete infarction (100%) (score 4).

We also assessed the neuronal pathological changes in acquired digital images saved as uncompressed 24-bit RGB TIFF files in the software program AnalySIS (Olympus Soft Imaging System GmbH, Munster, Germany), performing quantitative analysis of neuronal damage in the karyopyknotic areas. The neurons of cortical cerebral region, cerebellar region, hippocampus, and hypothalamus were counted in 10 different high-powered fields (HPF, 400×), and 3 to 5 serial sections of each sample were used to perform the counting as described in [84]. The field size was 0.24 μm^2^.

We used four criteria for estimation of the edema: a pale myelin, sieve-like appearance of myelinated areas, dilation of perivascular and pericellular spaces, and vacuolar appearance of the neuropil of gray matter. Edema was graded as heavy, moderate, slight, or no edema (score 0–3) [84].

We estimated hemorrhage as percentage of affected brain area. Intraventricular hemorrhage was noted as present or absent.

#### 4.9.2. Lung Histology

The same scoring system as in the previous studies [8,9,10,11,12,13,14,15,16,17,18,19,20,21,22,23,24,25] was used to grade renal (i.e., the degeneration of Bowman’s space and glomeruli, degeneration of the proximal and distal tubules, vascular congestion, and interstitial edema), liver (i.e., vacuolization of hepatocytes and pyknotic hepatocyte nuclei, activation of Kupffer cells, and enlargement of sinusoids) and heart (i.e., dilatation and congestion of blood vessels within the myocardium and coronary arteries) histology. Each specimen was scored using a scale ranging from 0 to 3 (0: none, 1: mild, 2: moderate, and 3: severe) for each criterion, and a final histology score was determined (0: none, 1: mild, 2: moderate, and 3: severe).

#### 4.9.3. Gastrointestinal Histology

As in previous studies [8,9,10,11,12,13,14,15,16,17,18,19,20,21,22,23,24,25], we used a histologic scoring scale adapted from Chui and coworkers [85] for the intestinal tissue damage scoring 0–5 (normal to severe) in three categories (mucosal injury, inflammation, hyperemia/hemorrhage) for a total score of 0 to 15, as described by Lane and coworkers [86]. Illustratively, the assessment included morphologic features of mucosal injury (i.e., different grades of epithelial lifting, villi denudation, and necrosis), inflammation (i.e., focal to diffuse according to lamina propria infiltration or subendothelial infiltration), and hyperemia/hemorrhage (i.e., focal to diffuse according to lamina propria or subendothelial localization).

### 4.10. Oxidative Stress

At the end of the experiment, oxidative stress in the collected tissue samples (brain, heart, lung, liver, kidney, stomach, small and large intestine, and blood) was assessed by quantifying the thiobarbituric acid-reactive species (TBARS) as malondialdehyde (MDA) [9,10,12,13,14,19,20]. The tissue samples were homogenized in PBS (pH 7.4) containing 0.1 mM butylated hydroxytoluene (BHT) (TissueRuptor, Qiagen, Valencia, CA, USA) and sonicated for 30 s in an ice bath (Ultrasonic Bath, Branson, MI, USA). Trichloroacetic acid (TCA, 10%) was added to the homogenate, the mixture was centrifuged at 3000 rpm for 5 min, and the supernatant was collected. Then, 1% TBA was added, and the samples were boiled (95 °C, 60 min). The tubes were then kept on ice for 10 min. Following centrifugation (14,000 rpm, 10 min), the absorbance of the mixture was determined at the wave length of 532 nm. The concentration of MDA was read from a standard calibration curve plotted using 1,1,3,3-tetraethoxypropane (TEP). The extent of lipid peroxidation was expressed as MDA using a molar extinction coefficient for MDA of 1.56 × 10^5^ mol/L/cm. The protein concentration was determined using a commercial kit. The results are expressed in nmol/mg of protein.

### 4.11. Statistical Analysis

Statistical analysis was performed using parametric one-way analysis of variance (ANOVA) with the Newman–Keuls post-hoc test or the non-parametric Kruskal–Wallis test and, subsequently, the Mann–Whitney U test to compare groups. Values are presented as means ± standard deviations (SDs) and as minimum/median/maximum values. To compare the frequency difference between groups, the chi-squared test or Fischer’s exact test was used. *p* < 0.05 was considered statistically significant.

## 5. Conclusions

In conclusion, such consistent therapy outcomes (resolved vascular and multiorgan failure; counteracted lesions and hemorrhage; almost-annihilated thrombosis, stasis, and arrhythmias; the counteraction of MDA value increases (peripherally and centrally); venous hypertension (intracranial (superior sagittal sinus), portal, and caval) and aortal hypotension being eliminated/attenuated; and advanced Virchow triad circumstances and decompression/reperfusion-severe occlusion/occlusion-like syndrome as a whole being counteracted) might be especially important. Conceptually, as in previous occlusion/occlusion-like syndromes [8,9,10,11,12,13,14,15,16,17,18,19,20,21,22,23,24,25] and in the present study, a particular cytoprotective agent ability might be resolving, at the general level, the reperfusion and therapy effect. Thus, rats with acute abdominal compartments and reperfusion, thereafter, are to be considered along with rats with Pringle maneuver [10], stroke [17], spinal cord compression [37,38], and ischemic/reperfusion ulcerative colitis [39]. In support of this fact, there have also been many beneficial effects on the brain [75,76,77,78,79], heart [87,88,89,90,91,92,93,94], lung [94,95,96,97], liver [63,98,99], kidney [100,101,102], and gastrointestinal [75,76,77,78,79] lesions in non-vascular studies. For BPC 157 therapy, these findings, along with the basic cytoprotection concept, posit that a cytoprotection mediator maintaining gastrointestinal tract integrity may translate into the particular therapy of other tissues and may be easily applied [1,2,3,4,5,6,30]. There, the resolved decompression–reperfusion occlusion/occlusion-like syndrome [8,9,10,11,12,13,14,15,16,17,18,19,20,21,22,23,24,25] goes as a prompt vascular defensive response (i.e., activation of the azygos vein direct blood flow delivery, noted in other occlusion/occlusion-like syndromes as well) even in very complex conditions. Thus, the envisaged vascular defensive response is effective at any time with BPC 157 therapy [8,9,10,11,12,13,14,15,16,17,18,19,20,21,22,23,24,25], and, upon injury, has been induced whatever the cause may be (ischemia or reperfusion), providing, also, the worst scenario of multiorgan injuries with the ischemia of intra-abdominal hypertension grade III and grade IV and its course thereafter, i.e., reperfusion after decompression. Finally, this conceptually goes with the evidence of the in situ hybridization and immunostaining of BPC 157 that has been found in the human fetus and adult gastrointestinal mucosa, the lung bronchial epithelium, the epidermal layer of the skin, and kidney glomeruli [2,103]. Possibly, BPC 157 may also have a regulatory physiologic role in bodily functions, based on the similar beneficial effects in other species (i.e., birds [96] and insects [104,105]). The final advantage is also a very safe BPC 157 profile (i.e., no adverse effects in clinical trials (ulcerative colitis, phase II), and in toxicological studies, LD1 could be not achieved) (for review see, i.e., [1,2,3,4,5,6,30,31,32,33,34], a point recently confirmed in a large study conducted by Xu and collaborators [106]. Together, these findings (for review see, i.e., [1,2,3,4,5,6,30,31,32,33,34]) might be suggestive of the further application of BPC 157 therapy in treating vascular injuries.

## Figures and Tables

**Table 1:** Blood pressures and thrombosis in rats at 60 min, 30 min, and 30 min following decompression after compression with intra-abdominal hypertension (25 mmHg/60 min; 30 mmHg/30 min; 40 mmHg/30 min). * p ˂ 0.05, *at least* vs. *control*.

	Blood Pressures and Thrombosis in Rats at 60 min, 30 min, and 25 min Following Decompression after Compression with Intra-abdominal Hypertension (25 mmHg/60 min; 30 mmHg/30 min; 40 mmHg/25 min)		
	60 min (25 mmHg)	30 min (30 mmHg)	30 min (40 mmHg)
	**Superior sagittal sinus pressure, mm Hg, means ± SD**		
Control	7 ± 1	8 ± 1	7 ± 1
BPC 157 10 μg/kg	−2 ± 1 *	−1 ± 1 *	−1 ± 1 *
BPC 157 10 ng/kg	−1 ± 1 *	−1 ± 1 *	−2 ± 1 *
	**Portal pressure, mm Hg, means ± SD**		
Control	17 ± 1	17 ± 2	18 ± 3
BPC 157 10 μg/kg	6 ± 1 *	5 ± 1 *	6 ± 1 *
BPC 157 10 ng/kg	6 ± 1 *	5 ± 1 *	6 ± 1 *
	**Caval pressure, mm Hg, means ± SD**		
Control	10 ± 2	11 ± 1	12 ± 2
BPC 157 10 μg/kg	5 ± 1 *	5 ± 1 *	5 ± 1 *
BPC 157 10 ng/kg	5 ± 1 *	5 ± 1 *	6 ± 1 *
	**Abdominal aorta pressure, mm Hg, means ± SD**		
Control	70 ± 5	65 ± 5	70 ± 5
BPC 157 10 μg/kg	100 ± 5 *	105 ± 7 *	98 ± 6 *
BPC 157 10 ng/kg	102 ± 7 *	98 ± 8 *	100 ± 7 *
	**Superior sagittal sinus, thrombus mass, g, means ± SD**		
Control	0.0019 ± 0.0004	0.0021 ± 0.0006	0.0042 ± 0.0008
BPC 157 10 μg/kg	0.0002 ± 0.0001 *	0.0006 ± 0.0002 *	0.0003 ± 0.0001 *
BPC 157 10 ng/kg	0.0001 ± 0.0001 *	0.0007 ± 0.0001 *	0.0005 ± 0.0002 *
	**Portal vein, thrombus mass, g, means ± SD**		
Control	0.0022 ± 0.0004	0.0035 ± 0.0006	0.0058 ± 0.0007
BPC 157 10 μg/kg	0.0005 ± 0.0001 *	0.0013 ± 0.0003 *	0.0002 ± 0.0001 *
BPC 157 10 ng/kg	0.0004 ± 0.0001 *	0.0010 ± 0.0002 *	0.0004 ± 0.0001 *
	**Inferior caval vein, thrombus mass, g, means ± SD**		
Control	0.0031 ± 0.0004	0.0082 ± 0.0004	0.0223 ± 0.0014
BPC 157 10 μg/kg	0.0008 ± 0.0004 *	0.0015 ± 0.0003 *	0.0029 ± 0.0005 *
BPC 157 10 ng/kg	0.0006 ± 0.0002 *	0.0010 ± 0.0004 *	0.0010 ± 0.0004 *
	**Abdominal aorta, thrombus mass, g, means ± SD**		
Control	0.0019 ± 0.0004	0.0022 ± 0.0005	0.0061 ± 0.0010
BPC 157 10 μg/kg	0.0005 ± 0.0001 *	0.0012 ± 0.0003 *	0.0011 ± 0.0004 *
BPC 157 10 ng/kg	0.0004 ± 0.0001 *	0.0010 ± 0.0004 *	0.0009 ± 0.0002 *

**Table 2:** The relative volume of the brain, peripheral blood vessels, and heart in rats at 60 min, 30 min, and 30 min following decompression after compression with intra-abdominal hypertension (25 mmHg/60 min; 30 mmHg/30 min; 40 mmHg/30 min). * p ˂ 0.05, *at least* vs. *control*.

	The Relative Volume of the Brain, Peripheral Blood Vessels, and Heart in Rats at 60 min, 30 min, and 30 min Following Decompression after Compression with Intra-Abdominal Hypertension (25 mmHg/60 min; 30 mmHg/30 min; 40 mmHg/30 min)		
	**60 min (25 mmHg)**	**30 min (30 mmHg)**	**30 min (40 mmHg)**
	Relative volume (control/treated) (%) of the brain, **means ± SD**		
BPC 157 10 μg/kg	110 ± 2 *	129 ± 5 *	120 ± 2 *
BPC 157 10 ng/kg	111 ± 2 *	125 ± 2 *	118 ± 2 *
	Relative volume (control/treated) (%) of the heart, **means ± SD**		
BPC 157 10 μg/kg	112 ± 3 *	141 ± 9 *	119 ± 6 *
BPC 157 10 ng/kg	116 ± 3 *	149 ± 9 *	122 ± 7 *
	Relative volume (control/treated) (%) of the azygos vein, **means ± SD**		
BPC 157 10 μg/kg	48 ± 5 *	48 ± 4 *	58 ± 5 *
BPC 157 10 ng/kg	46 ± 5 *	50 ± 6 *	54 ± 5 *
	Relative volume (control/treated) (%) of the inferior caval vein, **means ± SD**		
BPC 157 10 μg/kg	126 ± 5 *	158 ± 8 *	124 ± 5 *
BPC 157 10 ng/kg	124 ± 5 *	152 ± 9 *	128 ± 8 *
	Relative volume (control/treated) (%) of the superior mesenteric vein, **means ± SD**		
BPC 157 10 μg/kg	256 ± 9 *	269 ± 9 *	249 ± 9 *
BPC 157 10 ng/kg	266 ± 7 *	275 ± 8 *	255 ± 9 *
	Relative volume (control/treated) (%) of the brain, ex vivo **means ± SD**		
BPC 157 10 μg/kg	115 ± 3 *	119 ± 2 *	115 ± 4 *
BPC 157 10 ng/kg	117 ± 3 *	120 ± 2 *	117 ± 2 *

**Table 3:** ECG changes in rats after acute abdominal compartment, course during reperfusion and after therapy application. * p ˂ 0.05, *at least* vs. *control*.

	ECG Changes in Rats after Acute Abdominal Compartment, Course during Reperfusion and after Therapy Application					
Reperfusion assessment time (min)	0–15 min					
Time (min) after medication application	2 min			5 min		
	25 mmHg	30 mmHg	40 mmHg	25 mmHg	30 mmHg	40 mmHg
	**Heart frequency, beats/min, means ± SD**					
Control	300 ± 10	290 ± 10	280 ± 10	290 ± 10	280 ± 10	270 ± 10
BPC 157 10 μg/kg	370 ± 10 *	380 ± 9 *	375 ± 9 *	376 ± 10 *	378 ± 10 *	388 ± 8 *
BPC 157 10 ng/kg	378 ± 10 *	383 ± 8 *	376 ± 10 *	386 ± 9 *	375 ± 9 *	380 ± 10 *
**QTc intervals, ms, means ± SD**						
Control	170 ± 10	165 ± 5	165 ± 5	170 ± 10	165 ± 5	165 ± 5
BPC 157 10 μg/kg	200 ± 10 *	195 ± 5 *	199 ± 5 *	200 ± 10 *	195 ± 5 *	199 ± 5 *
BPC 157 10 ng/kg	200 ± 10 *	197 ± 5 *	196 ± 5 *	200 ± 10 *	197 ± 5 *	196 ± 5 *
**ST elevation, mV, means ± SD**						
Control	0.2 ± 0.1	0.2 ± 0.1	0.2 ± 0.1	0.2 ± 0.1	0.2 ± 0.1	0.2 ± 0.1
BPC 157 10 μg/kg	0 ± 0 *	0 ± 0 *	0 ± 0 *	0 ± 0 *	0 ± 0 *	0 ± 0 *
BPC 157 10 ng/kg	0 ± 0 *	0 ± 0 *	0 ± 0 *	0 ± 0 *	0 ± 0 *	0 ± 0 *
Reperfusion assessment time (min)	15–30 min					
Time (min) after medication application	15 min			25 min		
	25 mmHg	30 mmHg	40 mmHg	25 mmHg	30 mmHg	40 mmHg
	**Heart frequency, beats/min, means ± SD**					
Control	300 ± 10	260 ± 10	250 ± 10	290 ± 10	100 ± 10	90 ± 10
BPC 157 10 μg/kg	376 ± 10 *	370 ± 10 *	375 ± 9 *	386 ± 10 *	378 ± 10 *	375 ± 9 *
BPC 157 10 ng/kg	378 ± 10 *	373 ± 8 *	380 ± 9 *	380 ± 9 *	387 ± 8 *	380 ± 10 *
**QTc intervals, ms, means ± SD**						
Control	160 ± 10	163 ± 5	162 ± 5	160 ± 10	155 ± 5	159 ± 5
BPC 157 10 μg/kg	195 ± 5 *	200 ± 10 *	197 ± 5 *	198 ± 5 *	195 ± 5 *	200 ± 10 *
BPC 157 10 ng/kg	200 ± 8 *	198 ± 5 *	196 ± 5 *	199 ± 5 *	200 ± 10 *	196 ± 5 *
**ST elevation, mV, means ± SD**						
Control	0.2 ± 0.1	0.2 ± 0.1	0.2 ± 0.1	0.2 ± 0.1	0.2 ± 0.1	0.2 ± 0.1
BPC 157 10 μg/kg	0 ± 0 *	0 ± 0 *	0 ± 0 *	0 ± 0 *	0 ± 0 *	0 ± 0 *
BPC 157 10 ng/kg	0 ± 0 *	0 ± 0 *	0 ± 0 *	0 ± 0 *	0 ± 0 *	0 ± 0 *
Reperfusion assessment time (min)	30–60 min					
Time (min) after medication application	40 min			55 min		
	25 mmHg	30 mmHg	40 mmHg	25 mmHg	30 mmHg	40 mmHg
	**Heart frequency, beats/min, means ± SD**					
Control	250 ± 10	/	/	100 ± 10	/	/
BPC 157 10 μg/kg	386 ± 11 *	/	/	376 ± 10 *	/	/
BPC 157 10 ng/kg	388 ± 10 *	/	/	383 ± 9 *	/	/
**QTc intervals, ms, means ± SD**						
Control	160 ± 10	/	/	160 ± 10	/	/
BPC 157 10 μg/kg	200 ± 5 *	/	/	198 ± 5 *	/	/
BPC 157 10 ng/kg	196 ± 5 *	/	/	200 ± 5 *	/	/
**ST elevation, mV, means ± SD**						
Control	0.2 ± 0.1	/	/	0.2 ± 0.1	/	/
BPC 157 10 μg/kg	0 ± 0 *	/	/	0 ± 0 *	/	/
BPC 157 10 ng/kg	0 ± 0 *	/	/	0 ± 0 *	/	/

**Table 4:** MDA (brain, heart, lung, liver, kidney, blood, stomach, and small and large intestine) values in rats at 60 min, 30 min, and 25 min following decompression after compression with intra-abdominal hypertension (25 mmHg/60 min; 30 mmHg/30 min; 40 mmHg/30 min). * p ˂ 0.05, *at least* vs. *control*.

	MDA (Brain, Heart, Lung, Liver, Kidney, Blood, Stomach, Small and Large Intestine) or Macroscopic (Stomach) Values in Rats at 60 min, 30 min, and 30 min Following Decompression after Compression with Intra-Abdominal Hypertension (25 mmHg/60 min; 30 mmHg/30 min; 40 mmHg/25 min)		
	60 min (25 mmHg)	30 min (30 mmHg)	30 min (40 mmHg)
	**Brain nmol/mg protein, means** ± SD		
Control	8.7 ± 0.5	9 ± 0.5	10 ± 1.5
BPC 157 10 μg/kg	6.0 ± 0.3 *	6.4 ± 0.3 *	6.5 ± 0.5 *
BPC 157 10 ng/kg	5.5 ± 0.5 *	6.5 ± 0.5 *	7.5 ± 0.5 *
	**Heart nmol/mg protein, means** ± SD		
Control	52 ± 5	62 ± 5	66 ± 8
BPC 157 10 μg/kg	25 ± 7 *	28 ± 8 *	24 ± 5 *
BPC 157 10 ng/kg	22 ± 6 *	26 ± 6 *	29 ± 5 *
	**Lung nmol/mg protein, means** ± SD		
Control	62 ± 8	66 ± 7	60 ± 8
BPC 157 10 μg/kg	35 ± 8 *	38 ± 8 *	29 ± 5 *
BPC 157 10 ng/kg	34 ± 7 *	31 ± 6 *	35 ± 5 *
	**Liver, nmol/mg protein, means** ± SD		
Control	42 ± 5	45 ± 5	46 ± 3
BPC 157 10 μg/kg	30 ± 3 *	31 ± 3 *	30 ± 5 *
BPC 157 10 ng/kg	32 ± 3 *	32 ± 3 *	33 ± 5 *
	**Kidney nmol/mg protein, means** ± SD		
Control	25 ± 5	28 ± 5	46 ± 3
BPC 157 10 μg/kg	15 ± 3 *	17 ± 4 *	30 ± 5 *
BPC 157 10 ng/kg	17 ± 3 *	14 ± 3 *	33 ± 5 *
	**Blood (nmol/mg protein Means ± SD)**		
Control	55 ± 5	58 ± 8	60 ± 7
BPC 157 10 μg/kg	20 ± 3 *	18 ± 4 *	22 ± 5 *
BPC 157 10 ng/kg	16 ± 3 *	21 ± 5 *	25 ± 5 *
	**Stomach (nmol/mg protein Means ± SD)**		
Control	2.5 ± 0.5	3.5 ± 0.5	3.3 ± 0.5
BPC 157 10 μg/kg	1.0 ± 0.3 *	1.3 ± 0.3 *	1.5 ± 0.5 *
BPC 157 10 ng/kg	1.5 ± 0.2 *	1.4 ± 0.4 *	1.7 ± 0.5 *
	**Small intestine (nmol/mg protein Means ± SD)**		
Control	3.5 ± 0.5	3.3 ± 0.5	3.6 ± 0.7
BPC 157 10 μg/kg	1.0 ± 0.4 *	1.1 ± 0.3 *	1.2 ± 0.3 *
BPC 157 10 ng/kg	1.3 ± 0.2 *	1.5 ± 0.4 *	1.3 ± 0.4 *
	**Large intestine (nmol/mg protein Means ± SD)**		
Control	2.8 ± 0.5	3.1 ± 0.5	3.3 ± 0.7
BPC 157 10 μg/kg	1.0 ± 0.4 *	1.3 ± 0.3 *	1.4 ± 0.3 *
BPC 157 10 ng/kg	1.1 ± 0.2 *	1.2 ± 0.4 *	1.5 ± 0.4 *

**Table 5:** Lesions, scored microscopically (heart, lung, liver, kidney, stomach, small and large intestine), or macroscopically (stomach), in rats at 60 min, 30 min, and 30 min following decompression after compression with intra-abdominal hypertension (25 mmHg/60 min; 30 mmHg/30 min; 40 mmHg/30 min). * p ˂ 0.05, *at least* vs. *control*.

	Lesions, Scored Microscopically (Heart, Lung, Liver, Kidney, Stomach, Small and Large Intestine), or Macroscopically (Stomach), in Rats at 60 min, 30 min, and 30 min Following Decompression after Compression with Intra-Abdominal Hypertension (25 mmHg/60 min; 30 mmHg/30 min; 40 mmHg/30 min)		
	60 min (25 mmHg)	30 min (30 mmHg)	30 min (40 mmHg)
	**Heart (scored 0–3, Min/Med/Max)**		
Control	3/3/3	3/3/3	3/3/3
BPC 157 10 μg/kg	0/0/0 *	0/0/0 *	0/0/0 *
BPC 157 10 ng/kg	0/0/0 *	0/1/1 *	0/1/1 *
	**Lung (scored 0–3, Min/Med/Max)**		
Control	3/3/3	3/3/3	3/3/3
BPC 157 10 μg/kg	0/0/0 *	0/0/0 *	0/0/0 *
BPC 157 10 ng/kg	0/0/0 *	0/0/0 *	0/0/0 *
	**Liver (scored 0–3, Min/Med/Max)**		
Control	3/3/3	3/3/3	3/3/3
BPC 157 10 μg/kg	0/0/0 *	0/1/1 *	0/1/1 *
BPC 157 10 ng/kg	0/0/0 *	0/1/1 *	0/1/1 *
	**Kidney (scored 0–3, Min/Med/Max)**		
Control	3/3/3	3/3/3	3/3/3
BPC 157 10 μg/kg	0/0/0 *	0/1/1 *	0/1/1 *
BPC 157 10 ng/kg	0/0/0 *	0/1/1 *	0/1/1 *
	**Stomach (sum of longest diameters, mm, means ± SD)**		
Control	3/3/3	3/3/3	3/3/3
BPC 157 10 μg/kg	0/0/0 *	0/0/0 *	0/0/0 *
BPC 157 10 ng/kg	0/0/0 *	0/0/0 *	0/0/0 *
	**Stomach (scored 0–15, Min/Med/Max)**		
Control	3/3/3	3/3/3	3/3/3
BPC 157 10 μg/kg	0/0/0 *	0/0/0 *	0/0/0 *
BPC 157 10 ng/kg	0/0/0 *	0/0/0 *	0/0/0 *
	**Small intestine (scored 0–15, Min/Med/Max)**		
Control	3/3/3	3/3/3	3/3/3
BPC 157 10 μg/kg	0/0/0 *	0/0/0 *	0/0/0 *
BPC 157 10 ng/kg	0/0/0 *	0/0/0 *	0/0/0 *

**Table 6:** Lesions, scored microscopically (cerebrum, cerebellum, hypothalamus, hippocampus) in rats at 60 min, 30 min, and 30 min following decompression after compression with intra-abdominal hypertension (25 mmHg/60 min; 30 mmHg/30 min; 40 mmHg/30 min). Min/Med/Max, means ± SD, * p ˂ 0.05, *at least*, vs. *control.* # combined score (0–8)–semiquantitative neuropathological scoring system; the sum of affected areas with infarction and karyopyknotic cells.

	Lesions, Scored Microscopically Cerebrum, Cerebellum, Hypothalamus, and Hippocampus in Rats at 60 min (i), or 30 min (ii, iii) Following Decompression after Compression with Intra-Abdominal Hypertension (25 mmHg/60min (i); 30 mmHg/30min (ii); 40 mmHg/30min (iii)		
	60 min (25 mmHg)	30 min (30 mmHg)	30 min (40 mmHg)
	**Cerebrum (scored 0–8, Min/Med/Max) #**		
Control	2/3/3	3/3/3	3/3/3
BPC 157 10 μg/kg	0/1/1 *	0/1/1 *	0/1/1 *
BPC 157 10 ng/kg	0/1/1 *	0/1/1 *	0/1/1 *
	Neuronal damage in the karyopyknotic areas, %, means ± SD (10 HPF, 400×)		
Control	56 ± 5	64 ± 5	69 ± 5
BPC 157 10 μg/kg	14 ± 1 *	18 ± 1 *	15 ± 1 *
BPC 157 10 ng/kg	16 ± 1 *	14 ± 1 *	17 ± 1 *
	Hemorrhage (% of total area)		
Control	20 ± 3	30 ± 3	35 ± 3
BPC 157 10 μg/kg	2 ± 1 *	5 ± 2 *	5 ± 1 *
BPC 157 10 ng/kg	2 ± 1 *	6 ± 2 *	7 ± 1 *
	Edema (scored 0–3, Min/Med/Max)		
Control	2/3/3	3/3/3	3/3/3
BPC 157 10 μg/kg	0/1/1 *	0/1/1 *	0/1/1 *
BPC 157 10 ng/kg	0/1/1 *	0/1/1 *	0/1/1 *
	**Cerebellum (scored 0–8, Min/Med/Max)**		
Control	2/2/2	2/2/2	3/3/3
BPC 157 10 μg/kg	0/1/1 *	0/1/1 *	0/1/1 *
BPC 157 10 ng/kg	0/1/1 *	0/1/1 *	0/1/1 *
	Neuronal damage in the karyopyknotic areas, %, means ± SD (10 HPF, 400×)		
Control	55 ± 5	61 ± 4	73 ± 5
BPC 157 10 μg/kg	12 ± 3 *	16 ± 2 *	24 ± 3 *
BPC 157 10 ng/kg	15 ± 3 *	14 ± 2 *	22 ± 4 *
	Hemorrhage (% of total area)		
Control	0 ± 0	0 ± 0	0 ± 0
BPC 157 10 μg/kg	0 ± 0	0 ± 0	0 ± 0
BPC 157 10 ng/kg	0 ± 0	0 ± 0	0 ± 0
	Edema (scored 0–3, Min/Med/Max)		
Control	2/3/3	2/3/3	3/3/3
BPC 157 10 μg/kg	0/1/1 *	0/1/1 *	0/1/1 *
BPC 157 10 ng/kg	0/1/1 *	0/1/1 *	0/1/1 *
	**Hippocampus (scored 0–8, Min/Med/Max)**		
Control	2/3/3	2/3/3	2/3/3
BPC 157 10 μg/kg	0/1/1 *	0/1/1 *	0/1/1 *
BPC 157 10 ng/kg	0/1/1 *	0/1/1 *	0/1/1 *
	Neuronal damage in the karyopyknotic areas, %, means ± SD (10 HPF, 400×)		
Control	24 ± 3	25 ± 3	43 ± 5
BPC 157 10 μg/kg	2 ± 1 *	4 ± 1 *	12 ± 2 *
BPC 157 10 ng/kg	3 ± 1 *	3 ± 1 *	14 ± 2 *
	Hemorrhage (% of total area)		
Control	0 ± 0	0 ± 0	0 ± 0
BPC 157 10 μg/kg	0 ± 0	0 ± 0	0 ± 0
BPC 157 10 ng/kg	0 ± 0	0 ± 0	0 ± 0
	Edema (scored 0–3, Min/Med/Max)		
Control	2/2/3	2/2/3	2/3/3
BPC 157 10 μg/kg	0/0/0 *	0/0/0 *	0/0/0 *
BPC 157 10 ng/kg	0/0/0 *	0/0/0 *	0/0/0 *
	**Hypothalamus (scored 0–8, Min/Med/Max)**		
Control	3/3/3	3/3/3	3/3/3
BPC 157 10 μg/kg	0/1/1 *	0/1/1 *	0/1/1 *
BPC 157 10 ng/kg	0/1/1 *	0/1/1 *	0/1/1 *
	Neuronal damage in the karyopyknotic areas, %, means ± SD (10 HPF, 400×)		
Control	83 ± 4	96 ± 4	94 ± 6
BPC 157 10 μg/kg	21 ± 2 *	32 ± 2 *	43 ± 3 *
BPC 157 10 ng/kg	24 ± 2 *	35 ± 2 *	40 ± 3 *
	Hemorrhage (% of total area)		
Control	20 ± 2	25 ± 5	25 ± 2
BPC 157 10 μg/kg	0 ± 0 *	0 ± 0 *	0 ± 0 *
BPC 157 10 ng/kg	0 ± 0 *	0 ± 0 *	0 ± 0 *
	Edema (scored 0–3, Min/Med/Max)		
Control	2/3/3	3/3/3	3/3/3
BPC 157 10 μg/kg	0/1/1 *	0/1/1 *	0/1/1 *
BPC 157 10 ng/kg	0/1/1 *	0/1/1 *	0/1/1 *

## Data Availability

The data presented in this study are available on request from the corresponding author.
